# *Pelargonium graveolens* essential oil against clinical *Candida* and reference isolates: insights into virulence inhibition and therapeutic perspectives

**DOI:** 10.3389/ffunb.2026.1868001

**Published:** 2026-06-11

**Authors:** Chahrazed Benzaid, Manel Lina Djendi, Sarra Touaibia, Lina Raghed Klaiaia, Ahlam Kihal, Lazhari Tichati, Amel Soussa, Mahmoud Rouabhia

**Affiliations:** 1 Laboratory of Microbiology and Molecular Biology (LMMB), Department of Biochemistry, Sciences Faculty, Badji Mokhtar-Annaba University, Annaba, Algeria; 2 Department of Biochemistry, Environmental Research Center, Annaba, Algeria; 3 Groupe de Recherche en Écologie Buccale, Faculty of Dentistry, Université Laval, Quebec, QC, Canada

**Keywords:** anti-virulence, biofilm, *Candida*, geranium essential oil, phospholipase, secreted aspartyl protease, selectivity index

## Abstract

**Background:**

The rising incidence of *Candida*-associated infections and persistent antifungal resistance, driven by biofilm formation, highlight the need for alternative therapies. Essential oils offer promise as multi-targeted natural antifungals. This study evaluates the antifungal activity of *Pelargonium graveolens* essential oil (PGEO), obtained by hydrodistillation of aerial parts from the Edough region (Annaba, Algeria), against various *Candida* species and its effects on human cells.

**Methods:**

PGEO (yield: 0.23 ± 0.04% w/w) was characterized by GC-MS. Its antifungal activity was tested against five clinical *Candida* isolates (*C. albicans*, *C. kefyr*, *C. krusei (syn. Pichia kudriavzevii)*, *C. lusitaniae*, *C. tropicalis*) and reference strain *C. albicans* ATCC 10231. Effects on virulence enzymes (secreted aspartyl protease, phospholipase, esterase, hemolysin) were assessed via Pz/Hz indices on solid media. Anti-biofilm activity was assessed using crystal violet staining and metabolic viability assays. Cytotoxicity was evaluated in HEK-293 human embryonic kidney cells.

**Results:**

PGEO comprised mainly citronellol (28.68%), geraniol (5.35%), isomenthone (5.44%), and linalool (3.15%). It showed broad-spectrum antifungal activity (minimum inhibitory concentrations: 0.312–2.5 mg/mL; minimum fungicidal concentration/minimum inhibitory concentration ratios: 2), including against fluconazole-resistant *C. krusei*. At sub-inhibitory concentrations (minimum inhibitory concentration/4 to minimum inhibitory concentration/2); PGEO inhibited enzyme secretion by 21.30 to 65.00% depending on enzyme class and species ranging from 21.30% (esterase, *C. krusei*) to 65.00% (hemolysin, *C. albicans* ATCC 10231), reducing *Candida* adhesion and invasion. PGEO inhibited biofilm formation (48.7–97.8%) and metabolic activity (43.2–82.1% reduction at minimum inhibitory concentration). Cytotoxicity in HEK-293 cells yielded a CC_50_ of 3.99 ± 0.12 mg/mL, with selectivity indices of 1.59–12.79 (SI = CC_50_/MIC).

**Conclusions:**

The essential oil of *Pelargonium graveolens* exhibits potent fungicidal, antibiofilm, and multi-target antivirulence activities against clinically relevant *Candida* species, including fluconazole-resistant *C. krusei*, while maintaining acceptable cytotoxicity toward normal mammalian cells (CC_50_ = 3.99 ± 0.12 mg/mL; SI: 1.59–12.79). Its simultaneous inhibition of growth, key virulence enzymes, and biofilm formation at sub-inhibitory concentrations positions PGEO as a promising natural candidate for topical or adjunctive treatment of biofilm-associated candidiasis, pending *in vivo* validation.

## 1 Introduction

Invasive and superficial infections caused by *Candida* species are considered a serious global public health threat. According to current estimates from the mycological literature, fungal infections are responsible for approximately 3.8 million deaths worldwide each year, predominantly affecting immunocompromised individuals, patients in intensive care and post-surgical units, as well as those with medical devices such as central venous catheters and prosthetic joints (World Health Organization (WHO), 2022**;** Talapko et al., 2021**;** Amann et al., 2025).

In addition to biofilm production, *Candida* species also produce virulence enzymes, such as secreted aspartyl proteases (SAPs), phospholipases, esterases, and hemolysins. The functions of those virulence factors include adhesion, tissue invasion, nutrient acquisition, and evasion of the host immune response. Interestingly, *Candida* biofilms are also resistant to most antifungal agents, with resistance up to 1,000-fold greater than that of their planktonic counterparts, which explains treatment failures and relapse cases (Ramage et al., 2023**;** Li et al., 2025). Resistance to first-line antifungal drugs (azoles, echinocandins, amphotericin B) is emerging. It is detected in a significant number of clinical isolates, often belonging to non-albicans *Candida* species (*C. krusei (syn. Pichia kudriavzevii), C. tropicalis, C. parapsilosis, C. auris*). The spread of hospital strains carrying fluconazole-resistant clones necessitates the evaluation of alternative therapeutic approaches.

In light of the limitations of existing antifungal therapies — including their narrow spectrum, fungistatic rather than fungicidal activity, and increasing resistance burden — plant-derived essential oils (EOs) have emerged as promising alternative or adjunctive candidates for the management of *Candida* infections, with a low risk of developing resistance, and preferably low toxicity to human beings and the environment (Nazzaro et al., 2017).

The constituents in EOs, such as monoterpenes, sesquiterpenes, and phenylpropanoids, could disrupt fungal membranes, interfere with the expression of virulence factors, inhibit the adhesion of fungal propagules to surfaces, and modulate the expression of key genes involved in pathogenicity (Hou and Huang, 2024).

*Pelargonium graveolens* is a fragrant greenhouse crop distributed worldwide, mainly in North Africa. Essential oils obtained from the leaves of *P.graveolens* are rich in citronellol, geraniol, linalool, and menthone. Citronellol oil, commonly known as rose geranium oil, has been studied for its pharmacological activities. The results obtained have demonstrated fungistatic activity against a wide variety of fungal species, including human pathogenic dermatophytes and *Candida albicans* (Dzamic et al., 2014**;** Mahboubi and Valian, 2019**;** Giongo et al., 2016). Moreover, studies conducted on oil have revealed anti-inflammatory and antioxidant activity. However, most previous studies are often limited to basic antifungal activity without a simultaneous assessment of anti-virulence and anti-biofilm effects and, above all, cytotoxicity towards normal human cells.

The evaluation of cytotoxicity in the human embryonic kidney cell line (HEK-293) is particularly relevant because these cells are widely used as a standard model for assessing the selectivity and toxic potential of natural antifungal agents (Sadeghi et al., 2013**;** Asker and Al Haidar, 2024). A high selectivity index (SI) between antifungal activity and cytotoxicity in HEK-293 cells is essential for further development as a topical or adjuvant treatment.

The present study aimed to provide the first simultaneous evaluation of antifungal, antibiofilm, and antivirulence activities combined with cytotoxicity assessment in HEK-293 cells, for an Algerian PGEO against clinical *Candida* isolates including fluconazole-resistant strains.

## 2 Material and methods

### 2.1 Plant material and extraction

Fresh aerial parts (leaves and young stems) of *Pelargonium graveolens* L’Her. (*Geraniaceae*) were collected at the full flowering stage from the Edough region (Annaba, north-eastern Algeria, 36°52’N, 7°39’E), at an altitude of ~700 m above sea level, in May 2023.

The taxonomic identification of the species was carried out by Prof. Tarek Hamel (University of Annaba). A voucher specimen was deposited in the herbarium of Badji Mokhtar-Annaba University under accession number Pg 19-25: (Prof. Gérard de Bélair’s herbarium).

The essential oil was extracted by hydrodistillation from 100 g of air-dried, finely ground plant material. The operation was conducted using a Clevenger-type apparatus, in accordance with the recommendations of the European Pharmacopeia (Council of Europe, 2023) and based on the protocol described by Elyemni et al. (2019), with optimized operating parameters.

The yield was expressed as a percentage of dry plant material according to the equation:

Yield (%) = (mass of essential oil/mass of dry plant material) × 100.

### 2.2 Chemical analysis of essential oil

The chemical composition of the essential oil was determined by GC-FID and GC–MS. The identification of the constituents was based on comparisons of linear retention indices (RIs) obtained on columns with complementary polarities and mass spectra with data from the literature and reference spectral libraries. Where available, co-injections with authentic standards were used to confirm compound identification. Linear retention indices (LRI) were calculated for all identified compounds relative to a homologous series of n-alkanes (C_8_–C_28_), using the Van den Dool and Kratz equation (Van Den Dool and Kratz, 1963), and compared with published values from the literature (Adams, 2007**;** National Institute of Standards and Technology (NIST), 2017**;** Olascuaga-Castillo et al., 2024**;** Benzaid et al., 2021).

GC-FID analyses were performed on a MASTER GC DANI system equipped with a split/splitless injector, an autosampler, and an FID detector. Separation was carried out on an HP-5MS capillary column (30 m × 0.25 mm, 0.25 μm film thickness; 5% phenyl–95% dimethylpolysiloxane). The oven temperature program was as follows: 60 °C (8 min), ramp at 2 °C/min to 240 °C, followed by a 10-min isothermal hold. The injector and detector temperatures were 250 °C and 260 °C, respectively. Nitrogen was used as the carrier gas at a flow rate of 0.5 mL/min. A volume of 0.2 μL was injected in split mode (50:1). Electron ionization (EI) was performed at 70 eV, and mass spectra were acquired in the range m/z 35–450.Mass spectra were acquired under standard analytical conditions.

### 2.3 Evaluation of antifungal activity

The antifungal activity of *Pelargonium graveolens* essential oil (PGEO) was evaluated *in vitro* against: *C. albicans, C. kefyr, C. krusei, C. lusitaniae*, and *C. tropicalis.* The clinical isolates, derived from fecal samples of patients hospitalized in the medical intensive care unit, were provided by the medical microbiology laboratory at Annaba University Hospital (Algeria). At the same time, the reference strain *C. albicans* ATCC 10231 was obtained from the Pasteur Institute of Algeria.

Antifungal activity was determined using the agar diffusion method as described by Benzaid et al. (2019) according to CLSI guidelines with modifications for essential oils (Clinical and Laboratory Standards Institute (CLSI), 2018).

Fungal inocula were prepared from 18–24 h cultures and adjusted to an optical density of 0.08–0.1 at 600 nm (corresponding to approximately 1–5 × 10^6^ CFU/mL, equivalent to a 0.5 McFarland standard), measured spectrophotometrically for each species independently. Suspensions were uniformly spread onto Sabouraud dextrose agar (SDA, Merck Millipore) using sterile cotton swabs in three directions to ensure a homogeneous lawn. All species were processed in parallel under identical conditions (Li et al., 2023; Gaur et al., 2023). Sterile paper discs (6 mm diameter) were impregnated with 10 µL of PGEO and then placed on the surface of the inoculated media. Plates were incubated at 37 °C for 48 h (as recommended by CLSI M44-A2 guidelines for yeasts in disk-diffusion assays), in aerobic conditions. Inhibition zone diameters (DZIs, mm) were measured using a digital caliper, including the diameter of the disk (6 mm), from the inner edges of the inhibition zone on the agar surface. DMSO was used as a negative control, while Fluconazole (25 µg/disc) served as a positive control. All assays were performed in triplicate.

### 2.4 Determination of minimum inhibitory concentrations and minimum fungicidal concentrations

Minimum inhibitory concentrations (MIC) and minimum fungicidal concentrations (MFC) were determined by broth microdilution according to standardized protocols the Clinical and Laboratory Standards Institute (Clinical and Laboratory Standards Institute (CLSI), 2023**;** Gaur et al., 2023**;** Duarte et al., 2005). Fungal inocula were adjusted to a 0.5 McFarland standard (1 × 10^8^ CFU/mL), then diluted in Sabouraud broth (Merck Millipore) to achieve a final concentration of 1–2 × 10³ CFU/mL.

Serial 2-fold dilutions of the essential oil were prepared in sterile 96-well microplates. Each well contained 100 µL of essential oil solution and 100 µL of fungal inoculum, for a final volume of 200 µL.

Experimental controls included a growth control (medium + inoculum), a sterility control (medium alone), a solvent control, and a positive control (Fluconazole). After incubation at 37 °C for 48 h, fungal growth was assessed by measuring optical density at 600 nm. The MIC was defined as the lowest concentration at which no detectable growth was observed.

To determine the MFC, aliquots from wells showing no apparent growth were sub-cultured onto Sabouraud agar (Merck Millipore) and incubated for 48 h. The MFC corresponds to the lowest concentration resulting in a ≥99.9% reduction of the initial inoculum.

### 2.5 Inhibition of biofilm formation

The capacity of PGEO to inhibit *Candida* biofilm formation was evaluated using a co-incubation assay in polystyrene tubes, adapted from Negreiros et al. (2016). Fungal suspensions were prepared in Sabouraud broth (Merck Millipore) and adjusted to an optical density of 0.4 at 600 nm (approximately 1 × 10^7^ CFU/mL). Equal volumes (500 µL) of PGEO solution and fungal suspension were mixed to yield final PGEO concentrations corresponding to MIC, MIC/2, MIC/4, and MIC/8, and incubated under static conditions at 37 °C for 24 h to allow simultaneous cell adhesion and biofilm development.

At the end of incubation, the tubes were stained with crystal violet (0.1%, w/w) for 45 min. After removing excess dye, the fixed crystal violet was solubilized using an ethanol–acetone mixture (1:3, v/v). Biofilm biomass was quantified by measuring absorbance at 570 nm using a SECOMAM spectrophotometer. The percentage of biofilm formation inhibition was determined using the following equation:

% inhibition = [(Abs_control − Abs_test)/Abs_control] × 100.

### 2.6 Evaluation of biofilm metabolic activity (XTT assay)

Biofilm metabolic activity was quantified using the XTT assay, (2,3-bis(2-methoxy-4-nitro-5-sulfophenyl)-2H-tetrazolium-5-carboxanilide). The assay was performed according to Seddiki et al. (2010)**;** Kuhn et al. (2003) and Pierce et al. (2008), with modifications.

A stock solution of XTT (Sigma Chemical Co., St. Louis, MO) was prepared (0.5mg/mL) in sterile PBS and filtered. Menadione solution (1 mM in acetone) was added as an electron-coupling agent (final concentration: 1 µM). A total of 100 µL of XTT-menadione solution was added to each well. Plates were incubated in the dark at 37 °C for 2–3 h.

The reduction of XTT produce an orange-colored formazan, which was quantified by measuring absorbance at 490 nm using a microplate reader.

The percentage of inhibition of metabolic activity was calculated from the mean optical densities of the treated samples and the control, according to the following equation:

% inhibition = [(Abs_control − Abs_test)/Abs_control] × 100.

### 2.7 Evaluation of the inhibitory activity on virulence-associated enzymes

The virulence of *Candida* spp. is strongly associated with the secretion of hydrolytic enzymes, including phospholipases, secreted aspartyl proteases (SAPs), esterases, and hemolysins, all of which contribute to host tissue invasion and immune evasion (Naglik et al., 2003**;** Schaller et al., 2005). Four reference agar-diffusion assays were performed to evaluate the inhibitory potential of the PGEO on these enzymatic activities at a sub-inhibitory concentration (¼ MIC).

#### 2.7.1 phospholipase activity

Phospholipase activity was assessed using the egg yolk precipitation method described by Price et al. (1982) and subsequently by Kantarcioglu and Yücel (2002).

The base medium was prepared by dissolving Sabouraud dextrose agar (SDA, 13 g), NaCl (11.7 g), and CaCl_2_ (0.11 g) in 184 ml of distilled water, followed by autoclaving at 120 °C for 20 minutes. After cooling to 50 °C, 20 ml of egg yolk supernatant sterilized by filtration — obtained by centrifugation at 3,000 × g for 10 minutes at 4 °C — was added aseptically. CaCl_2_ is an essential component of the medium because it promotes the visible precipitation of fatty acids released by phospholipase activity. PGEO was incorporated into the medium at ¼ MIC before pouring the plates. The inoculates were prepared at a concentration of 10^8^ cells/mL in sterile physiological saline, and 10 µL aliquots were dispensed into the center of the agar surface. The plates were incubated at 37 °C for 72 hours.

#### 2.7.2 Assay of secreted aspartyl protease activity

SAP activity was assessed using the bovine serum albumin (BSA) (Himedia) hydrolysis method described by Rüchel (1981), as modified by Naglik et al. (2003). SAPs are acidic aspartyl proteases that constitute primary virulence determinants enabling tissue invasion in *Candida* (Naglik et al., 2003). The active protease degrades the BSA incorporated into the medium, creating a transparent clearing zone on an otherwise opaque surface.

The test medium (pH 3.5, the optimal pH for SAP activity) was prepared by dissolving the following in 60 ml of distilled water: MgSO_4_ (0.04 g), K_2_HPO_4_ (0.5 g), NaCl (1 g), yeast extract (0.2 g), glucose (4 g), and BSA (0.5 g). The pH was adjusted to 3.5 with 0.1 N HCl. As BSA undergoes irreversible denaturation during autoclaving, the complete solution was sterilized by filtration through 0.22 µm membranes (Millex^®^-GV, Merck Millipore, Burlington, MA, USA) and added aseptically to 140 ml of molten agar equilibrated at 52 °C. PGEO was added at ¼ MIC concentrations before pouring the plates. Inocula at 10^6^ cells/mL were dispensed (10 µL per spot), and the plates were incubated at 37 °C for 7 days, as SAP production by *Candida* occurs gradually under these conditions (Naglik et al., 2003).

#### 2.7.3 Esterase activity assay

Esterase activity was assessed using the Tween 80 (Sigma- France) opacity method described by Sierra (1957), as applied to *Candida* by Buzzini and Martini (2002).

The culture medium was prepared by dissolving peptone (10 g), NaCl (5 g), CaCl_2_ (0.1 g), and agar (15 g) in 1 L of distilled water, followed by autoclaving at 121 °C for 20 min. After cooling to 50 °C, Tween 80 (5 mL) was pre-sterilized by filtration through 0.22 µm membranes. The PGEO was incorporated at ¼MIC concentration before pouring. Inocula at 10^6^–10^7^ cells/mL were spotted (10 µL per spot), and the plates were incubated at 37 °C for 10 days, a duration sufficient to allow full development of Ca²^+^ precipitate halos.

#### 2.7.4 Hemolysin activity assay

Hemolysin activity was assessed using the sheep blood agar method described by Luo et al. (2001). SDA was prepared and autoclaved at 121 °C for 20 min. After cooling to 45–48 °C, 7% (v/v) fresh defibrinated sheep blood was added aseptically, together with the PGEO at ¼ MIC concentrations. Inoculate at 10^6^ cells/mL was spotted (10 µL per spot), and the plates were incubated at 37 °C for 48 h. *Candida* hemolysins—including candidalysin encoded by ECE1 and various SAP-related hemolytic proteins lyse erythrocytes and contribute to vascular invasion (Moyes et al., 2016). Complete lysis (β-hemolysis) produces a translucent, clear halo, partial oxidative lysis (α-hemolysis) produces a greenish discoloration, and the absence of any halo (γ-reaction) indicates a non-hemolytic phenotype.

#### 2.7.5 Measurement parameters

Enzyme activity was quantified using the Pz index (for phospholipase, SAP and esterase) or Hz index (for hemolysin) as defined by Price et al. (1982): Pz or Hz = d_c/(d_c + d_h) where d_c is the colony diameter and d_h is the diameter of the halo.

% Inhibition = [(Index_control − Index_treated)/Index_control] × 100.

Activity was classified as follows:

Pz = 1.00, negative (no activity);

0.64 < Pz < 1.00, weak (+);

0.40 ≤ Pz ≤ 0.64, moderate (++);

Pz < 0.40, strong (+++) (Price et al., 1982**).**

Data are expressed as the mean ± standard deviation (SD) of three independent experiments.

### 2.8 Cytotoxicity assay

Cytotoxicity was assessed using the MTT assay according to the protocol by Saleh et al. (2014), adapted for lipophilic natural extracts, using human embryonic kidney cells (HEK-293, ATCC CRL-1573) as a model of normal human cells. Briefly, cells were seeded at 1 × 10^4^ cells/well into 96-well plates and incubated for 48 h to allow adhesion. The cells were then treated with different concentrations of essential oil (7.8–8,000 µg/mL) for 48 h. DMSO (1%, v/v) served as the negative control, whereas doxorubicin (10 µM) (Sigma-Aldrich, D1515) was used as the positive control. After treatment, the cells were washed twice with PBS, then an MTT solution (0.5 mg/mL) (Sigma-France) was added and incubated for 4 hours. The resulting formazan crystals were dissolved in acidified isopropanol, and absorbance was measured at 570 nm with a reference at 630 nm using a microplate reader (FilterMax F5, Molecular Devices). Cell viability (%) was calculated relative to untreated controls Nine wells per concentration (3 technical × 3 biological replicates) were used. The half-maximal cytotoxic concentration (CC_50_) was determined by nonlinear regression analysis using GraphPad Prism 9.0 (GraphPad Software, San Diego, CA, USA). The selectivity index (SI) was calculated as the ratio of the CC_50_/MIC.

### 2.9 Statistical analysis

All assays were performed in triplicate (n = 3 biological replicates). Data are expressed as mean ± SD. Nonlinear dose-response curves (CC_50_) were fitted using four-parameter logistic regression in GraphPad Prism 9.0(GraphPad Software, San Diego, CA, USA).

## 3 Results

### 3.1 Chemical composition of *Pelargonium graveolens* essential oil

Hydrodistillation of 100 g of dried aerial parts of *Pelargonium graveolens* using a Clevenger-type apparatus yielded greenish-yellow essential oil with a yield of 0.23 ± 0.04% (w/w, dry weight basis).

**Figure 1:**
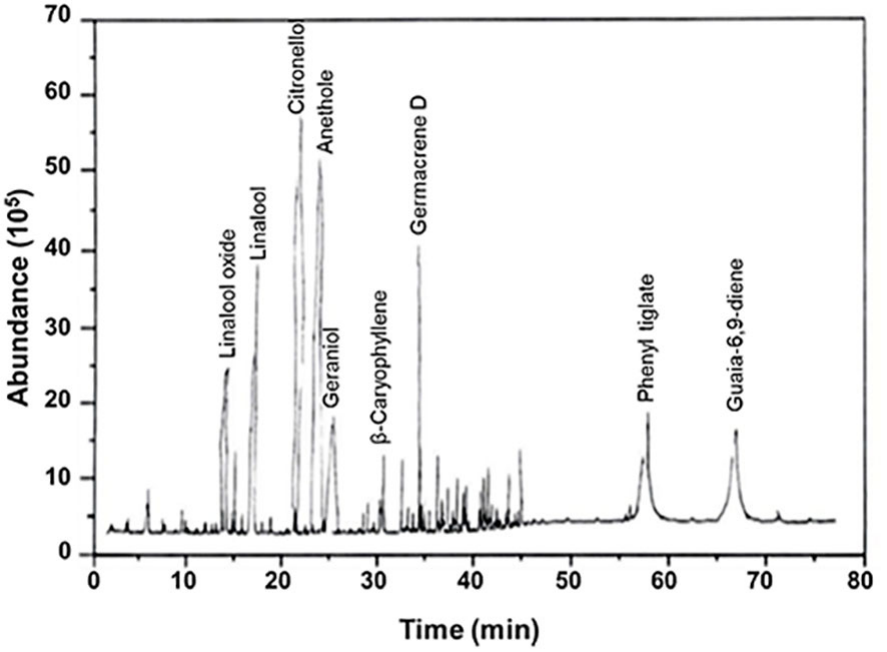
GC–MS chromatogram of the essential oil obtained from the aerial parts of *Pelargonium graveolens*.

GC and GC–MS analyses identified 58 compounds, representing 81.5% of the total oil composition, from 70 detected peaks (**Table 1**; **Figure 1**). The chemical profile is predominantly composed of monoterpenols (37.72%), followed by oxidized monoterpenes (11.48%), sesquiterpene hydrocarbons (11.41%), phenylpropanoids/aromatic esters (9.66%), and monoterpene esters (8.48%). whilst aromatic compounds (0.17%), monoterpene hydrocarbons (1.95%), oxygenated sesquiterpenes (0.48%), and other minor oxygenated compounds (0.16%) are present in lower proportions.

**Table 1:** Chemical composition of *Pelargonium graveolens* essential oil from the Edough region (Annaba, Algeria), determined by GC-FID and GC–MS analyses.

N°	RT	Compound	LRI	%	Class
1	2.50	3-Methyl-1-butanol	736	0.05	Other oxygenated
2	4.20	(Z)-3-Hexen-1-ol	855	0.11	Other oxygenated
3	6.19	α-Pinene	938	0.61	Monoterpene HC
4	8.02	*cis*-Rose oxide	1110	0.10	Oxidized monoterpene
5	8.16	β-Myrcene	991	0.24	Monoterpene HC
6	8.26	2-Menthene	1006	0.09	Monoterpene HC
7	8.68	α-Phellandrene	1004	0.06	Monoterpene HC
8	9.54	*p*-Cymene	1025	0.17	Aromatic monoterpene
9	9.85	Dihydromyrcenol	1069	0.09	Monoterpenol
10	10.16	(E)-β-Ocimene	1050	0.14	Monoterpene HC
11	10.64	δ-3-Carene	1011	0.10	Monoterpene HC
12	11.86	*cis*-Linalool oxide	1074	0.27	Oxidized monoterpene
13	**13.41**	**Linalool**	**1098**	**3.15**	**Monoterpenol**
**14**	**13.92**	***trans*-Linalool oxide**	**1088**	**2.22**	**Oxidized monoterpene**
**15**	**16.25**	**Menthone**	**1153**	**2.53**	**Oxidized monoterpene**
**16**	**16.93**	**Isomenthone**	**1162**	**5.44**	**Oxidized monoterpene**
17	17.88	Menthol	1171	0.15	Monoterpenol
18	18.37	γ-Terpineol	1199	0.30	Monoterpenol
**19**	**21.48**	**Citronellol**	**1228**	**28.68**	**Monoterpenol**
20	21.68	(Z)-β-Ocimene	1037	0.26	Monoterpene HC
**21**	**22.86**	**Geraniol**	**1255**	**5.35**	**Monoterpenol**
22	23.92	Citronellyl formate	1274	0.76	Monoterpene ester
23	24.07	Linalyl formate	1218	0.11	Monoterpene ester
**24**	**24.25**	**(E)-Anethole**	**1284**	**4.31**	**Phenylpropanoid**
**25**	**25.37**	**Geranyl formate**	**1300**	**1.56**	**Monoterpene ester**
26	27.92	*α-caryophyllene)*	1454	0.43	Sesquiterpene HC
27	29.45	α-Cubebene	1351	0.39	Sesquiterpene HC
28	29.99	β-Bourbonene	1387	1.03	Sesquiterpene HC
29	30.32	Geranyl acetate	1381	0.16	Monoterpene ester
30	30.51	β-Elemene	1390	0.16	Sesquiterpene HC
31	32.02	(E)-β-Caryophyllene	1418	1.00	Sesquiterpene HC
32	32.60	*Epi_*Bicyclosesquiphellandrene	1422	0.10	Sesquiterpene HC
33	33.27	α-Guaiene	1439	0.45	Sesquiterpene HC
34	33.65	Isolongifolene	1407	0.52	Sesquiterpene HC
**35**	**33.86**	**Citronellyl propionate**	**1453**	**1.08**	**Monoterpene ester**
36	34.46	Allo-aromadendrene	1460	0.26	Sesquiterpene HC
37	35.30	Calamenene	1521	0.26	Sesquiterpene HC
38	35.71	Germacrene D	1481	1.02	Sesquiterpene HC
39	35.96	β-Selinene	1490	0.16	Sesquiterpene HC
40	36.57	*trans*-Rose oxide	1125	0.80	Oxidized monoterpene
41	36.95	α-Muurolene	1500	0.13	Sesquiterpene HC
42	37.70	γ-Muurolene	1478	0.16	Sesquiterpene HC
43	38.01	α-Copaene	1376	0.12	Sesquiterpene HC
44	38.28	δ-Cadinene	1522	0.99	Sesquiterpene HC
45	38.75	Citronellyl butyrate	1531	0.88	Monoterpene ester
46	40.15	Geranyl butyrate	1562	0.48	Monoterpene ester
47	40.55	Geranial *(citral b)*	1267	0.12	Oxidized monoterpene
48	40.72	Caryophyllene oxide	1581	0.15	Oxygenated sesquiterpene
49	41.66	Neryl propionate	1490	0.12	Monoterpene ester
50	41.91	β-Eudesmol	1649	0.14	Oxygenated sesquiterpene
51	42.77	τ-Cadinol	1640	0.10	Oxygenate sesquiterpene
52	43.13	α-Cadinol	1652	0.09	Oxygenated sesquiterpene
53	43.54	*p*-Menth-1-ene	1019	0.45	Monoterpene HC
54	44.49	Neryl isovalerate	1583	0.50	Monoterpene ester
**55**	**57.97**	**Germacrene B**	**1559**	**2.02**	**Sesquiterpene HC**
**56**	**58.36**	**2-Phenylethyl tiglate**	**1591**	**5.35**	**Aromatic ester**
**57**	**67.71**	**Geranyl tiglate**	**1696**	**4.07**	**Monoterpene ester**
**58**	**67.84**	**Guaia-6,9-diene**	**1442**	**1.40**	**Sesquiterpene HC**

Chemical composition (%) of *Pelargonium graveolens* essential oil from the Edough region (Annaba, Algeria) analyzed by GC-FID and GC–MS. The Table presents retention times (RT), compound names, and relative percentages. Major compounds are highlighted in bold. Total identified compounds represent 81.5% of the oil. See **Figure 1** for the GC–MS chromatogram..

LRI calculated according to Van Den Dool and Kratz (1963) on HP-5 column relative to a homologous series of n-alkanes (C_8_–C_28_); LRI ref = literature values (Adams et al., 2007**;** National Institute of Standards and Technology (NIST), 2017).

The chemical profile is dominated by monoterpenols, with citronellol accounting for the largest proportion (28.68%), followed by geraniol (5.35%) and linalool (3.15%). This dominance reflects a chemical signature typical of *Pelargonium graveolens* essential oils, characterized by a high content of oxygenated compounds (citronellyl formate, isomenthone) with strong biological activity. Oxidized monoterpenes are mainly represented by isomenthone (5.44%), followed by menthone (2.53%) and linalool oxide (2.22%). Sesquiterpene hydrocarbons constitute another significant fraction, with β-bourbonene (1.03%), germacrene D (1.02%), δ-cadinene (0.99%), germacrene B (2.02%), and guaia-6,9-diene (1.40%) as the principal identified components.

Phenylpropanoids and aromatic esters are represented by 2-phenylethyl tiglate (5.35%) and (E)-anethole (4.31%), collectively accounting for 9.66% of the oil. Monoterpene esters are dominated by geranyl tiglate (4.07%), geranyl formate (1.56%), citronellyl propionate (1.08%), suggesting a significant contribution of these esterified derivatives to the overall profile.

Finally, minor oxygenated compounds, such as geranial (0.12%), dihydromyrcenol (0.09%), and 10-epi-γ-eudesmol (0.05%), were identified. Aromatic compounds are poorly represented overall, with p-cymene (0.17%) as the main detected constituent.

### 3.2 Antifungal activity

**Figure 2:**
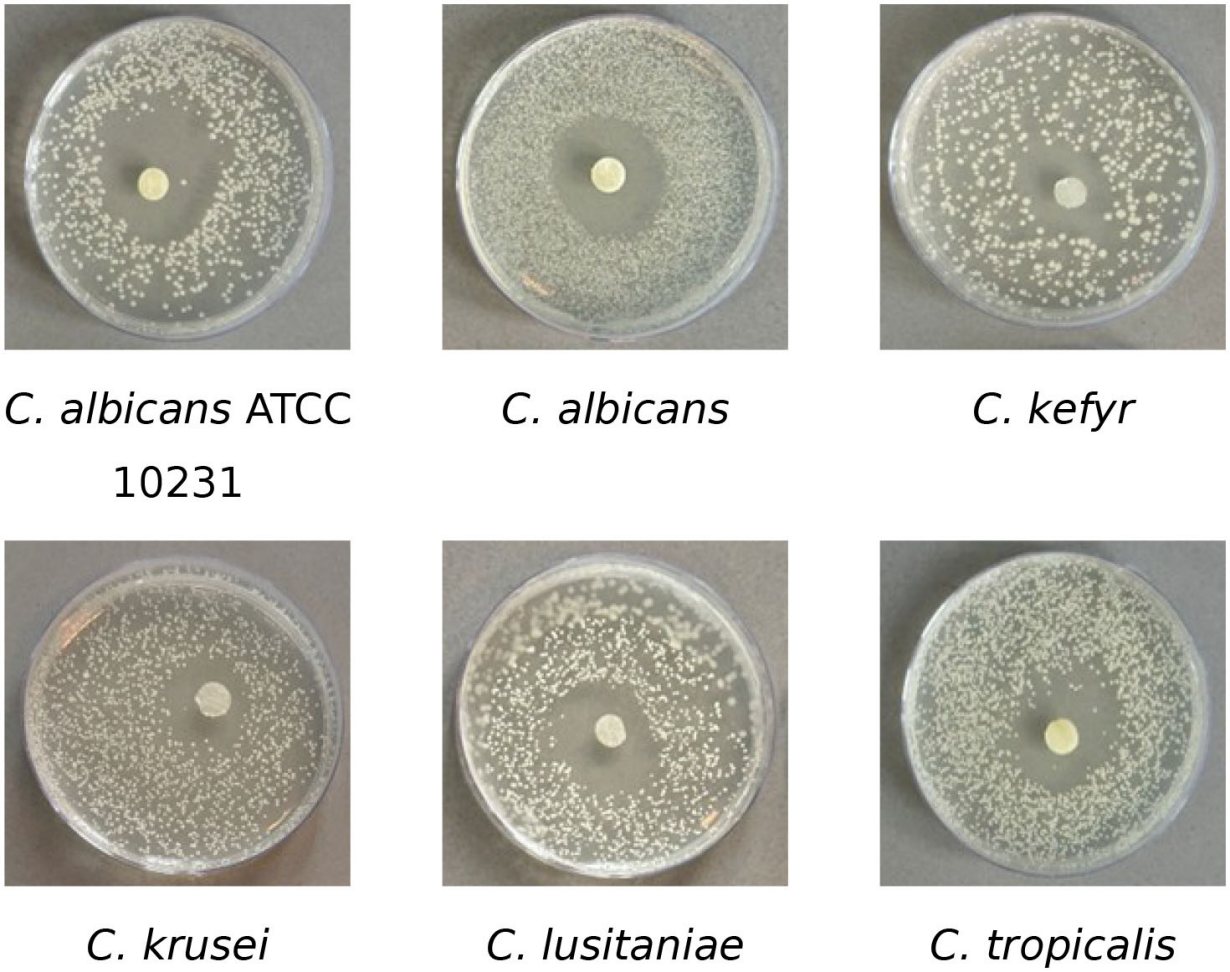
Inhibition of *Candida* spp. growth on solid medium by *Pelargonium graveolens* essential oil (PGEO) assessed by agar disk diffusion.

The antifungal activity of PGEO was evaluated using complementary approaches, including measurement of inhibition zone diameters (DZI, mm) as a qualitative indicator and determination of minimum inhibitory concentrations (MIC, µg/mL) and minimum fungicidal concentrations (MFC) as quantitative parameters of antifungal activity. These last two criteria allow, respectively, for the estimation of the inhibitory and lethal effects of the essential oil against the tested strains. All results are presented in **Table 2**; **Figure 2**.

**Table 2:** Antifungal activity of the essential oil from *Pelargonium graveolens* (PGEO) against *Candida* spp. Strains.

Strains	DZI (mm) PGEO 10 µL	MIC (mg/mL)	MFC (mg/mL)	Ratio MFC/MIC	Effect	DZI Fluconazole 25 µg/disc	DZI DMSO 1%	Growth control
*C.albicans* ATCC 10231	24.5 ± 1.2	0.312	0.625	2	Fungicidal	28.0 ± 0.8	0 mm	+++(confluent)
*C.albicans*	22.8 ± 0.9	0.625	1.25	2	Fungicidal	26.5 ± 1.1	0 mm	+++
*C.kefyr*	19.3 ± 1.0	1.25	2.5	2	Fungicidal	22.0 ± 0.7	0 mm	+++
*C.krusei*	15.7 ± 0.8	2.5	5.0	2	Fungicidal	0 mm	0 mm	+++
*C.lusitaniae*	21.6 ± 1.1	0.625	1.25	2	Fungicidal	25.0 ± 0.9	0 mm	+++
*C. tropicalis*	23.1 ± 0.7	0.625	1.25	2	Fungicidal	24.5 ± 1.0	0 mm	+++

DZI, inhibition zone diameter (mm); MIC, minimum inhibitory concentration; MFC, minimum fungicidal concentration; PGEO, essential oil from *Pelargonium graveolens* L’Hér. For each species, untreated control plates (DMSO 1% solvent control) confirmed the formation of a dense, confluent growth lawn (denoted +++ in **Table 2**), validating the inoculum viability and the absence of solvent-related inhibition for all six tested species. Results are expressed as mean ± standard deviation (n = 3). Fluconazole (25 µg/disc) was used as a positive control according to CLSI M27 guidelines, 2023. DMSO (final concentration ≤ 1%) served as solvent and negative control. +++, confluent growth (viability control). The difference was not statistically significant (p > 0.9999).

The MFC/MIC ratio was used to classify the mode of action of PGEO: a ratio ≤ 4 indicates a fungicidal effect, while a ratio > 4 indicates a fungistatic effect (Gaur et al., 2023**;** Floyd et al., 2024).

A fungicidal effect is preferred clinically, as it reduces the risk of relapse following treatment discontinuation.

Diameters of inhibition zones (DZIs, mm) produced by PGEO (10 µL/disk) against six *Candida* strains: *C. albicans* ATCC 10231 (reference strain), *C. albicans* (clinical isolate), *C. kefyr*, *C. krusei*, *C. lusitaniae*, and *C. tropicalis*. Plates were incubated at 37 °C for 48 h on Sabouraud dextrose agar. For each species, untreated control plates (DMSO 1% solvent control) confirmed the formation of a dense, confluent growth lawn (denoted +++ in **Table 2**), validating the inoculum viability and the absence of solvent-related inhibition for all six tested species. Results are expressed as mean ± standard deviation (n = 3). (GraphPad Prism 9.0, GraphPad Software, San Diego, CA, USA). All assays were performed in triplicate across three independent biological replicates (n = 3, i.e., 9 total measurements per data point). Data are expressed as mean ± standard deviation (SD). Statistical comparisons were performed using two-way ANOVA followed by Tukey’s HSD *post-hoc* test (GraphPad Prism 9.0). Significance levels: ns = p > 0.05; * p < 0.05; ** p < 0.01; *** p < 0.001; **** p < 0.0001.

PGEO inhibited all six *Candida* strains. DZIs at 10 µL ranged from 15.7 ± 0.8 mm *C. krusei* to 24.5 ± 1.2 mm *C. albicans* ATCC 10231. The DMSO 1% negative control showed no inhibition zone (0 mm), validating the solvent’s neutrality. Fluconazole (positive control, 25 µg/disc) yielded DZIs of 24–28 mm for susceptible strains and 0 mm for *C. krusei* (intrinsic resistance confirmed). MIC values ranged from 0.312 mg/mL (*C. albicans* ATCC 10231) to 2.5 mg/mL (*C. krusei*). The MFC/MIC ratio was uniformly 2 for all strains, confirming a fungicidal mode of action (threshold ≤ 4; CLSI M27 guidelines, 2023) (Gaur et al., 2023; Floyd et al., 2024). PGEO thus exerts fungicidal activity against *C. krusei* despite its intrinsic fluconazole resistance.

The visual differences in colony density observed among the six *Candida* species in the disk-diffusion assay (**Figure 2**) are consistent with recognized interspecific growth characteristics. *C. albicans* (both strains) and *C. tropicalis* produce dense, opaque, creamy-white lawns with strong agar adhesion, yielding the most visually uniform plates. *C. kefyr* grows more rapidly but spreads more diffusely, resulting in a thinner lawn despite equivalent inoculum density. *C. lusitaniae* produces a smooth but less pigmented colony surface, and *C. krusei* is characterized by its flat, spreading, dry-surface morphotype, which can appear less dense in photographs but reaches the required inoculum density upon spectrophotometric adjustment (OD_600_ = 0.08–0.1; ~0.5 McFarland). These differences in colonial morphology and surface coverage are intrinsic to each species and do not compromise the standardization of the assay (Talapko et al., 2021; Ramage et al., 2023).

### 3.3 Inhibition of biofilm formation: crystal violet biomass assay and metabolic viability

**Figure 3:**
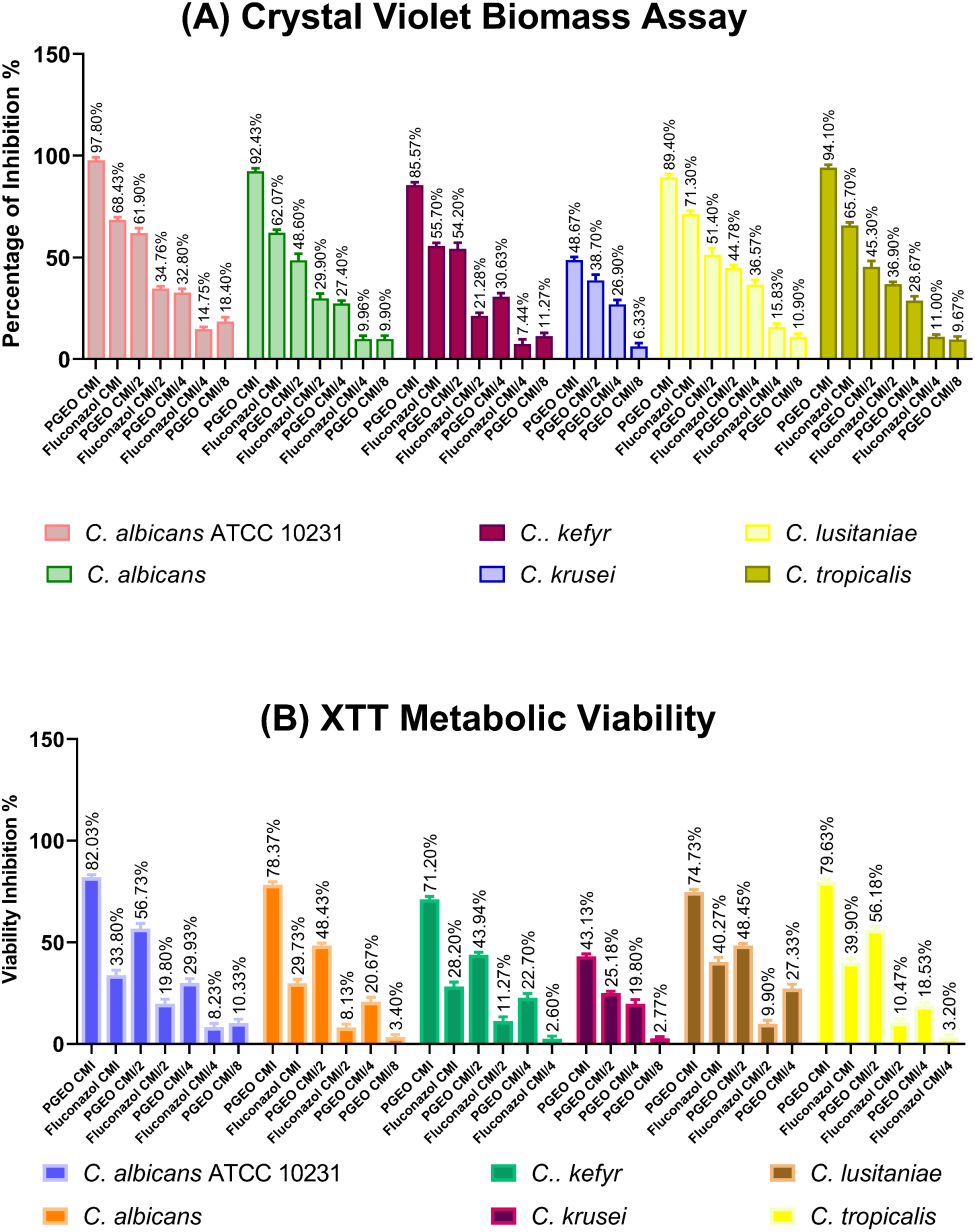
Antibiofilm activity of *Pelargonium graveolens* essential oil (PGEO) against six *Candida* species. **(A)** Inhibition of biofilm biomass (Crystal Violet assay). **(B)** Inhibition of metabolic activity (XTT assay). All assays were performed in triplicate across three independent biological replicates (n = 3, i.e., 9 total measurements per data point). Data are expressed as mean ± standard deviation (SD). Statistical comparisons were performed using two-way ANOVA followed by Tukey’s HSD *post-hoc* test (GraphPad Prism 9.0). Significance levels: ns, p > 0.05; ****p < 0.0001.

The inhibition of biofilm formation of PGEO against the same six *Candida* species was quantified using two independent and complementary assays: (A) crystal violet (CV) staining, which measures total biofilm biomass, and (B) 2,3-bis(2-methoxy-4-nitro-5-sulfophenyl)-2H-tetrazolium-5-carboxanilide) (XTT) reduction, which reflects the metabolic viability of sessile cells (**Figure 3**).

Both assays were performed at four PGEO concentrations (MIC, MIC/2, MIC/4, MIC/8) and compared against fluconazole at equivalent dilutions.

PGEO exhibited potent antibiofilm activity, reducing biofilm biomass (crystal violet assay) by 48.67 ± 1.55% to 97.80 ± 1.47% at MIC, with the highest inhibition observed against *C. albicans* ATCC 10231. The XTT assay confirmed a reduction in metabolic activity of sessile cells ranging from 43.20% to 82.10%.

XTT values at MIC ranged from 43.20 ± 1.40% (*C. krusei*) to 82.10 ± 1.40% (*C. albicans* ATCC 10231). The two assays yielded congruent, albeit not identical results — the systematically lower XTT values relative to CV at equivalent concentrations reflect the methodological distinction between bulk biomass measurement (CV) and real-time metabolic activity of viable sessile cells (XTT).

A clear, consistent dose-response relationship was observed across all species and both assays (**Figure 3**). Inhibition decreased progressively from MIC to MIC/8 without complete loss of activity, with residual inhibition of 6.33–18.40% (CV) and 0–10.33% (XTT) recorded at MIC/8.

A key finding was the significant antibiofilm effect against *C. krusei* (≈ 48.7% biomass and 43.1% metabolic inhibition at MIC), whereas fluconazole showed no activity against this strain. This differential response underscores the therapeutic potential of PGEO in fluconazole-resistant and biofilm-associated infections.

### 3.4 Inhibition of *Candida* virulence enzymes secretions

**Figure 4:**
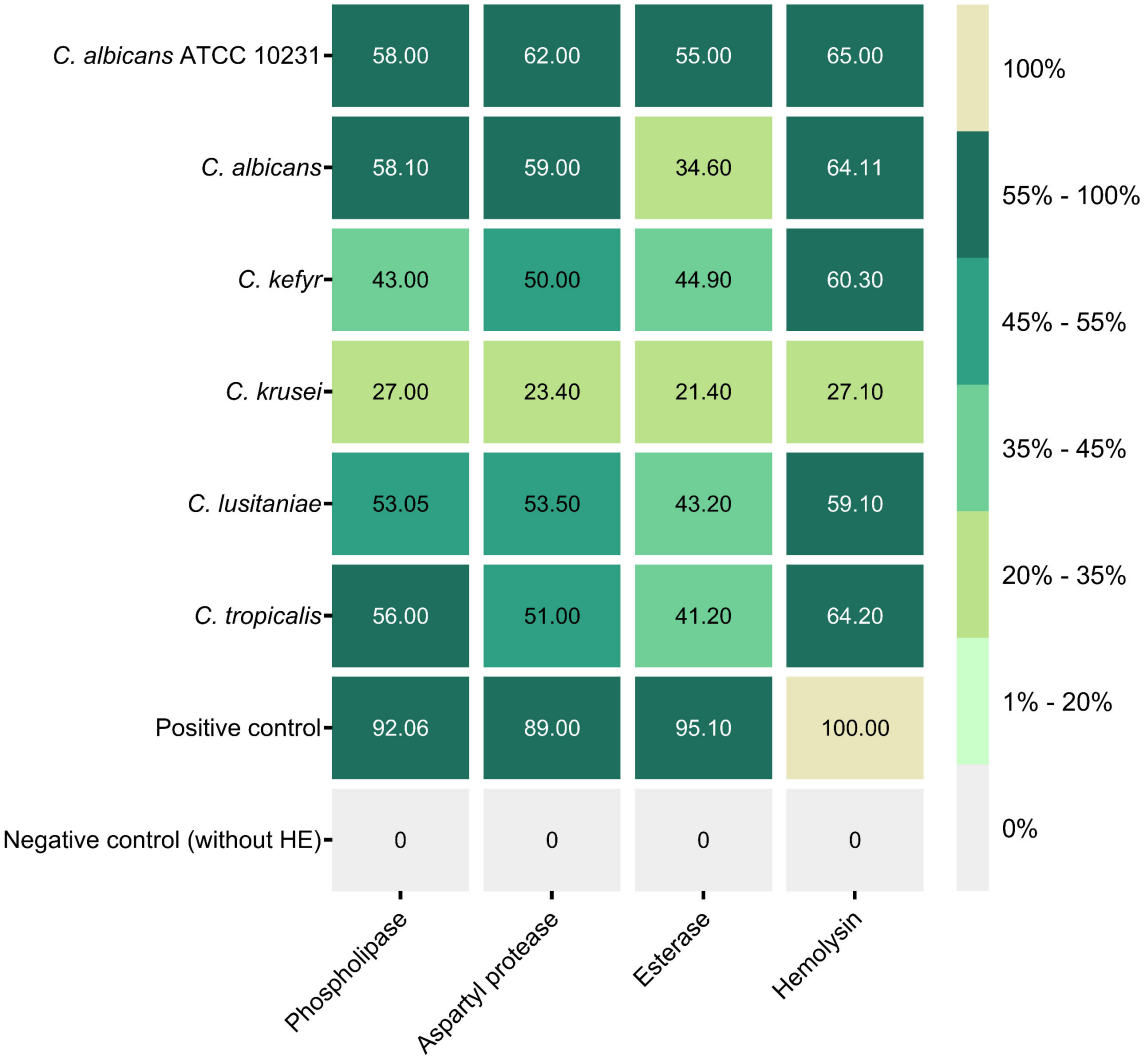
Comparative inhibition of *Candida* virulence enzyme secretion (phospholipase, SAP, esterase, hemolysin) by *Pelargonium graveolens* essential oil (PGEO) at ¼ MIC.

The effect of PGEO on four extracellular virulence enzymes—phospholipase, secreted aspartyl protease (SAP), esterase, and hemolysin—was assessed for all six *Candida* species using substrate-specific agar diffusion assays with a ¼ MIC concentration. Enzyme activity was quantified using the Pz index (phospholipase, SAP, esterase) and the Hz index (hemolysin), where lower values indicate stronger enzymatic activity; inhibition was expressed as the percentage reduction relative to untreated controls. Results are summarized in **Tables 3**–**6** , **Figure 4**.

**Table 3:** Inhibition of phospholipase activity in clinical and reference *Candida* isolates by *Pelargonium graveolens* essential oil (PGEO) at sub-inhibitory concentration (¼ MIC).

Strain	Pz_basal (untreated)	Pz_PGEO (¼ MIC)	Inhibition (%)	Post-treatment activity class
*C.albicans* ATCC 10231	0.550 ± 0.02	0.231 ± 0.01	58.00 ± 0.82	Strong (+++)
*C.albicans* (clinical)	0.570 ± 0.02	0.239 ± 0.01	58.03 ± 0.40	Strong (+++)
*C.kefyr*	0.700 ± 0.03	0.399 ± 0.02	43.00 ± 1.02	Strong (+++)
*C.krusei*	0.800 ± 0.02	0.584 ± 0.02	27.00 ± 1.11	Moderate (++)
*C.lusitaniae*	0.620 ± 0.02	0.291 ± 0.01	53.02 ± 0.80	Strong (+++)
*C.tropicalis*	0.580 ± 0.02	0.255 ± 0.01	56.00 ± 1.40	Strong (+++)
Reference inhibitor (EDTA,5 mM)	0.550 ± 0.02	0.985 ± 0.01	92.02 ± 0.82	Negative (−)
Solvent control (DMSO1%, v/v)	0.550 ± 0.02	0.553 ± 0.02	0.55 ± 0.30 (NS)	Strong (+++)
Untreated control	0.550 ± 0.02	—	0.00 ± 0.00	Strong (+++)

• Pz index (Price et al., 1982), d_⊂_/(d_⊂_ + d_c̲_), where d_⊂_ is the colony diameter and d_⊂̲_ is the precipitation halo diameter measured from the colony edge.

• Pz classification; Pz, 1.00 → negative (−); 0.64 < Pz < 1.00 → weak (+); 0.40 ≤ Pz ≤ 0.64 → moderate (++); Pz < 0.40 → strong (+++).

• Inhibition (%) = [(Pz_basal − Pz_treated)/Pz_basal] × 100.

• EDTA (5 mM) was used as the reference inhibitor since phospholipase activity strictly requires Ca²^+^ as a cofactor (Samaranayake et al., 2005).

• NS, not significant vs. untreated control (p <0,0001, two-way ANOVA followed by Tukey’s HSD).

• PGEO, *Pelargonium graveolens* essential oil; ¼ MIC, one-quarter of the species-specific minimum inhibitory concentration (see **Table 2**). Pz indices before and after treatment, percentage of inhibition, and post-treatment activity classification (mean ± SD; n = 3 independent experiments performed in triplicate).

**Table 4:** Inhibition of secreted aspartyl protease (SAP) activity in clinical and reference *Candida* isolates by PGEO at ¼ MIC.

Strain	Pz_basal (untreated)	Pz_PGEO (¼ MIC)	Inhibition (%)	Post-treatment activity class
*C.albicans* ATCC 10231	0.520 ± 0.02	0.198 ± 0.01	62.00 ± 1.20	Strong (+++)
*C.albicans* (clinical)	0.540 ± 0.02	0.221 ± 0.01	59.07 ± 1.70	Strong (+++)
*C.kefyr*	0.650 ± 0.03	0.324 ± 0.02	50.17 ± 1.76	Strong (+++)
*C.krusei*	0.820 ± 0.03	0.629 ± 0.02	23.30 ± 1.65	Moderate (++)
*C.lusitaniae*	0.600 ± 0.02	0.277 ± 0.01	53.83 ± 2.02	Strong (+++)
*C.tropicalis*	0.630 ± 0.02	0.308 ± 0.01	51.13 ± 1.60	Strong (+++)
Reference inhibitor (Pepstatin A, 10 µM)	0.520 ± 0.02	0.972 ± 0.01	89.23 ± 1.27	Negative (−)
Solvent control (DMSO 1%, v/v)	0.520 ± 0.02	0.524 ± 0.02	0.77 ± 0.45 (NS)	Strong (+++)
Untreated control	0.520 ± 0.02	—	0.00 ± 0.00	Strong (+++)

• Pz index (Price et al., 1982; Naglik et al., 2003), classification thresholds, and inhibition formula: see **Table 3**.

• Pepstatin A (10 µM) was used as a reference inhibitor of aspartic proteases (Naglik et al., 2003); it specifically targets the catalytic Asp32/Asp215 residues of *SAP*s.

• Assay conditions: BSA-supplemented medium at pH 3.5 (optimal for SAP activity); 7-day incubation at 37 °C (Naglik et al., 2003; Rüchel, 1981).

• NS, not significant vs. untreated control (p <0,0001, Tukey’s HSD).

• The lower susceptibility of *C. krusei* may reflect preferential expression of *SAP4–SAP6* isoforms, which are structurally less accessible to small-molecule terpene-mediated interference. Pz indices before and after treatment, percentage of inhibition, and post-treatment activity classification (mean ± SD; n = 3).

**Table 5:** Inhibition of esterase activity in clinical and reference *Candida* isolates by PGEO at ¼ MIC.

Strain	Ez_basal (untreated)	Ez_PGEO (¼ MIC)	Inhibition (%)	Post-treatment activity class
*C.albicans* ATCC 10231	0.580 ± 0.02	0.261 ± 0.01	55.00 ± 0.95	Strong (+++)
*C.albicans* (clinical)	0.720 ± 0.03	0.472 ± 0.02	34.47 ± 1.60	Moderate (++)
*C. kefyr*	0.680 ± 0.03	0.377 ± 0.02	44.60 ± 1.77	Strong (+++)
*C.krusei*	0.850 ± 0.03	0.669 ± 0.02	21.30 ± 1.55	Weak (+)
*C. lusitaniae*	0.700 ± 0.03	0.397 ± 0.02	43.23 ± 1.75	Strong (+++)
*C.tropicalis*	0.720 ± 0.03	0.423 ± 0.02	41.23 ± 1.65	Moderate (++)
Reference inhibitor (PMSF, 1 mM)	0.580 ± 0.02	0.987 ± 0.01	95.03 ± 1.40	Negative (−)
Solvent control (DMSO 1%, v/v)	0.580 ± 0.02	0.582 ± 0.02	0.34 ± 0.28 (NS)	Strong (+++)
Untreated control	0.580 ± 0.02	—	0.00 ± 0.00	Strong (+++)

• Ez index is calculated identically to the Pz index but applied to esterase opacity halos around colonies on Tween 80–calcium medium (Sierra, 1957; Buzzini and Martini, 2002). Classification thresholds (negative, weak, moderate, strong) are identical to those of Pz (Price et al., 1982).

• PMSF (phenylmethylsulfonyl fluoride, 1 mM) was used as a reference inhibitor of serine hydrolases including esterases (Buzzini and Martini, 2002).

• Assay conditions: Tween 80 (5 mL/L) supplemented agar with CaCl_2_ (0.1 g/L); 10-day incubation at 37 °C, allowing full development of Ca²^+^-fatty acid precipitate halos.

• NS, not significant vs. untreated control (p<0,0001, Tukey’s HSD).

• The lower esterase inhibition in the *C.albicans* clinical isolate (34.47%) compared with the reference strain (55.00%) may reflect overexpression of efflux pumps (*CDR1*/*CDR2*) reducing intracellular EO accumulation (De Sousa et al., 2020). Pz indices before and after treatment, percentage of inhibition, and post-treatment activity classification (mean ± SD; n = 3).

**Table 6:** Inhibition of hemolysin activity in clinical and reference *Candida* isolates by PGEO at ¼ MIC.

Strain	Hz_basal (untreated)	Hz_PGEO (¼ MIC)	Inhibition (%)	Post-treatment activity class
*C.albicans* ATCC 10231	0.480 ± 0.02	0.168 ± 0.01	65.00 ± 1.30	Strong (+++)
*C.albicans* (clinical)	0.510 ± 0.02	0.183 ± 0.01	64.10 ± 1.60	Strong (+++)
*C.kefyr*	0.550 ± 0.02	0.219 ± 0.01	60.27 ± 1.75	Strong (+++)
*C.krusei*	0.780 ± 0.03	0.568 ± 0.02	27.13 ± 1.55	Moderate (++)
*C.lusitaniae*	0.550 ± 0.02	0.225 ± 0.01	59.08 ± 1.83	Strong (+++)
*C.tropicalis*	0.500 ± 0.02	0.178 ± 0.01	64.31 ± 1.67	Strong (+++)
Reference inhibitor (heat-inactivation, 70 °C × 30 min)	0.480 ± 0.02	1.000 ± 0.00	100.00 ± 0.00	Negative (−)
Solvent control (DMSO 1%, v/v)	0.480 ± 0.02	0.483 ± 0.02	0.62 ± 0.31 NS)	Strong (+++)
Untreated control	0.480 ± 0.02	—	0.00 ± 0.00	Strong (+++)

• Hz index (Luo et al., 2001) = d_⊂_/(d_⊂_ + d_⊂̲_), where d_⊂_ is the colony diameter and d_⊂̲_ is the diameter of the hemolytic halo measured from the colony edge.

• Hz classification: Hz = 1.00 → no hemolysis (γ-reaction, −); 0.64 < Hz < 1.00 → weak hemolysis (+); 0.40 ≤ Hz ≤ 0.64 → moderate hemolysis (++); Hz < 0.40 → strong hemolysis (+++).

• Heat-inactivation of the inoculum (70 °C, 30 min) was used as the reference inhibitor since it abolishes protein-mediated hemolytic activity (notably candidalysin encoded by *ECE1* and SAP-related hemolytic factors); under these conditions no halo forms (d_⊂̲_ = 0), yielding Hz, 1.00 by definition.

• Assay conditions: Sabouraud dextrose agar supplemented with 7% (v/v) defibrinated sheep blood; 48 h incubation at 37 °C in 5% CO_2_.

• NS, not significant vs. untreated control (p <0,0001, Tukey’s HSD).

• Important correction notice: the previously reported value of Hz_basal = 0.00 for the positive control was a typographical error; mathematically, Hz cannot equal 0 since d_⊂_ > 0 (colony always present). The corrected value Hz = 1.00 reflects the absence of any hemolytic halo upon heat inactivation. Hz indices before and after treatment, percentage of inhibition, and post-treatment activity classification (mean ± SD; n = 3).

Assay validation. Positive enzyme controls confirmed robust baseline activity across all four assays (phospholipase: 92.02 ± 0.82%; SAP: 89.23 ± 1.27%; esterase: 95.03 ± 1.40%; hemolysin: 100.00 ± 0.00%). Negative controls (no essential oil) showed no inhibition (0.00 ± 0.00% for all enzymes and all strains), confirming assay specificity and the absence of spontaneous enzyme suppression.

All assays were performed in triplicate across three independent biological replicates (n = 3, i.e., 9 total measurements per data point). Data are expressed as mean ± standard deviation (SD). Statistical comparisons were performed using two-way ANOVA followed by Tukey’s HSD *post-hoc* test (GraphPad Prism 9.0). Significance levels: ns = p > 0.05; * p < 0.05; ** p < 0.01; *** p < 0.001; **** p < 0.0001.

PGEO inhibited phospholipase activity in all six strains, with inhibition ranging from 27.00 ± 1.11% (*C. krusei*) to 58.00 ± 0.82% (*C. albicans* ATCC 10231). The post-treatment Pz values for the two most susceptible strains — *C. albicans* ATCC 10231 (Pz = 0.231) and *C. albicans* clinical isolate (Pz = 0.239) — fell below 0.40, corresponding to strong inhibitory activity according to the classification of Price et al. (1982). *C. kefyr* (Pz = 0.399), *C. lusitaniae* (Pz = 0.291), and *C. tropicalis* (Pz = 0.255) also showed strong post-treatment activity. In contrast, *C. krusei* exhibited only moderate inhibition (Pz = 0.584; 0.40 ≤ Pz ≤ 0.64), consistent with its intrinsically lower basal phospholipase activity (baseline Pz = 0.800).

#### 3.4.1 Secreted aspartyl protease inhibition

SAP inhibition followed a similar pattern, with the highest values recorded for *C.albicans* ATCC 10231 (62.00 ± 1.20%; post-treatment Pz = 0.198) and the *C.albicans* clinical isolate (59.07 ± 1.70%; Pz = 0.221), both classified as strong inhibition (Pz < 0.40). Moderate-to-strong inhibition was observed for *C.lusitaniae* (53.83 ± 2.02%; Pz = 0.277), *C.tropicalis* (51.13 ± 1.60%; Pz = 0.308), and *C.kefyr* (50.17 ± 1.76%; Pz = 0.324). *C.krusei* again showed the lowest susceptibility (23.30 ± 1.65%; Pz = 0.629), remaining in the weak inhibition range (0.64 < Pz < 1.00).

#### 3.4.2 Esterase inhibition

Esterase was the least inhibited enzymatic activity overall, with inhibition ranging from 21.30 ± 1.55% (*C.krusei*; post-treatment Ez = 0.669) to 55.00 ± 0.95% (*C.albicans* ATCC 10231; Ez = 0.261). Notably, the *C.albicans* clinical isolate showed markedly lower esterase inhibition (34.47 ± 1.60%; Ez = 0.472) compared with the reference strain (55.00 ± 0.95%), suggesting strain-level variability in esterase susceptibility. *C.kefyr* (44.60 ± 1.77%), *C.lusitaniae* (43.23 ± 1.75%), and *C. tropicalis* (41.23 ± 1.65%) showed intermediate inhibition levels.

#### 3.4.3 Hemolysin inhibition

Hemolysin was the most consistently inhibited virulence factor across species. Inhibition ranged from 27.13 ± 1.55% (*C. krusei*; Hz = 0.568) to 65.00 ± 1.30% (*C. albicans* ATCC 10231; Hz = 0.168). Strong inhibition (Hz < 0.40) was recorded for *C.albicans* ATCC 10231 (Hz = 0.168), *C. albicans* clinical isolate (Hz = 0.183), *C.kefyr* (Hz = 0.219), *C.lusitaniae* (Hz = 0.225), and *C. tropicalis* (Hz = 0.178). *C.krusei* remained in the moderate range (Hz = 0.568).

### 3.5 Cytotoxicity assay

**Figure 5:**
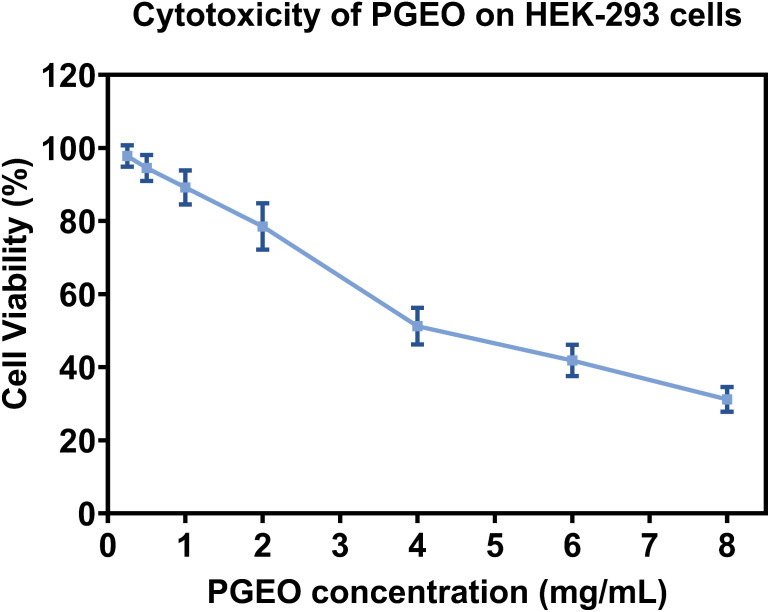
Dose-response curve of PGEO cytotoxicity on HEK-293 cells (MTT assay) during 48h exposure. All assays were performed in triplicate across three independent biological replicates (n = 3, i.e., 9 total measurements per data point). Data are expressed as mean ± standard deviation (SD). Statistical comparisons were performed using one-way ANOVA (GraphPad Prism 9.0).

To assess the safety profile of PGEO for potential therapeutic use, its cytotoxicity was evaluated on normal human embryonic kidney (HEK-293) cells using the MTT assay. Results are presented in **Table 7**, **Figure 5**.

**Table 7:** Dose-response cytotoxicity of PGEO on HEK-293 cells: cell viability (%) and ISO 10993–5 classification (mean ± SD, n = 3).

PGEO conc. (mg/mL)	Cell viability (% ± SD)	ISO 10993–5 classification
0.25	97.80 ± 2.94	Non-cytotoxic (> 75%)
0.50	94.50 ± 3.55	Non-cytotoxic (> 75%)
1.00	89.20 ± 4.66	Non-cytotoxic (> 75%)
2.00	78.50 ± 6.37	Non-cytotoxic (> 75%)
4.00	51.20 ± 5.02	Slightly cytotoxic (50–75%)
6.00	41.80 ± 4.29	Cytotoxic (< 50%)
8.00	31.20 ± 3.43	Cytotoxic (< 50%)
DMSO 1%	100.00 ± 3.80	Negative control (reference)
Doxorubicin 10 µM	8.50 ± 1.47	Positive cytotoxic control (validated)

ISO 10993-5 (2009) classification thresholds: > 75% non-cytotoxic; 50–75% slightly cytotoxic; < 50% cytotoxic.

The cytotoxicity profile of PGEO on HEK-293 cells was clearly dose-dependent, as assessed according to ISO 10993-5 (International Organization for Standardization (ISO), 2009). At low concentrations (0.25 to 2 mg/mL), cell viability remained above 75% in all cases (97.80%, 94.50%, 89.20%, and 78.50%, respectively), placing PGEO within the non-cytotoxic category throughout this range. Viability declined progressively at higher concentrations: at 4 mg/mL it reached 51.20% (slightly cytotoxic, 50–75%), and at 6 and 8 mg/mL it fell to 41.80% and 31.20%, respectively, both classified as cytotoxic (< 50%). The negative control (DMSO 1%) yielded 100.00 ± 3.80% viability, validating the cell model. The positive control (doxorubicin 10 µM) showed a viability of only 8.50 ± 1.47%, corresponding to >90% cell death and confirming the assay’s sensitivity. The CC_50_ of PGEO on HEK-293 cells, determined by four-parameter nonlinear regression (GraphPad Prism 9.0), was 3.99 ± 0.12 mg/mL (n = 18 wells, three independent experiments). This value represents the intrinsic cytotoxic potential of PGEO toward the human cell model, independent of the fungal strain. The corresponding selectivity indices (SI = CC_50_/MIC), reflecting the species-dependent therapeutic window, are reported in **Table 8**.

**Table 8:** CC_50_ of PGEO on HEK-293 cells, Selectivity Index (SI), and cytotoxicity controls (mean ± SD, n = 3 independent experiments).

Strain	MIC (mg/mL)	SI = CC_50_/MIC	Interpretation
*C.albicans* ATCC 10231	0.312	12.79	Excellent (SI > 10)
*C.albicans* (clinical)	0.625	6.38	Favorable (SI > 4)
*C.kefyr*	1.25	3.19	Moderate (2 < SI < 4)
*C.krusei*	2.50	1.59	Narrow (SI < 2)
*C.lusitaniae*	0.625	6.38	Favorable (SI > 4)
*C.tropicalis*	0.625	6.38	Favorable (SI > 4)
DMSO 1%	96.8–98.2% viability	—	Negative control (p = NS vs untreated)
Untreated cells	100.00 ± 0.00%	—	Baseline reference

CC_50_ (HEK-293) = 3.99 ± 0.12 mg/mL (pooled value, n = 18 wells). SI = CC_50_ (mg/mL)/MIC (mg/mL). SI interpretation criteria: SI > 10: excellent; SI > 4: favorable; 2 < SI < 4: moderate; SI < 2: narrow (Elizondo-Luévano et al., 2023). ISO 10993-5: viability > 75% at ≤ 2 mg/mL = non-cytotoxic..

The selectivity indices ranged from 1.59 (*C.krusei*) to 12.78 (*C.albicans* ATCC 10231). According to Elizondo-Luévano et al. (2023), PGEO demonstrated excellent selectivity against *C.albicans* ATCC 10231 (SI = 12.79; SI > 10), favorable selectivity against *C.albicans* (clinical isolate), *C.lusitaniae*, and *C.tropicalis* (SI = 6.38 for all three; SI > 4), moderate selectivity against *C.kefyr* (SI = 3.19; 2 < SI < 4), and a narrow therapeutic window against *C.krusei* (SI = 1.59; SI < 2). These findings confirm that PGEO is non-cytotoxic at concentrations ≤ 2 mg/mL (ISO 10993-5), a threshold that encompasses the MICs of the four most susceptible species tested (*C.albicans* ATCC 10231, *C.albicans* clinical isolate, *C.lusitaniae*, and *C.tropicalis***).**

## 4 Discussion

The present study provides the first integrated assessment of antifungal, antibiofilm, antivirulence, and cytotoxic activities of *Pelargonium graveolens* essential oil (PGEO) sourced from the Edough massif (north-eastern Algeria), against a clinically relevant panel of *Candida* species, including the intrinsically fluconazole-resistant *Pichia kudriavzevii* (formerly *Candida krusei*). Our results delineate a multitarget pharmacological profile of PGEO, with a clear hierarchy of activity across species and enzymatic targets, and a selectivity window that supports its further development as a topical or adjunctive anticandidal agent.

### 4.1 Chemical composition and chemotype attribution

The hydrodistillation yielded PGEO at 0.23 ± 0.04% (w/w, dry matter); this value is consistent with previous data, particularly those reported by Atailia and Djahoudi (2015) and Wei et al. (2022), who report yields ranging from 0.23% to 0.25%. This consistency suggests reproducibility of the extraction process. However, the essential oil yield remains inherently low and highly dependent on exogenous factors, such as plant material origin and experimental conditions (Dos Santos et al., 2024**;** Ling et al., 2022). The oil belongs to the citronellol-rich chemotype, typical of the Maghreb region. The major constituents were citronellol (28.68%), guaia-6,9-diene (5.94%), isomenthone (5.44%), 2-phenylethyl tiglate (5.35%) and geraniol (5.35%). These results are in agreement with previous Algerian reports (Atailia and Djahoudi, 2015; Boukhatem et al., 2013), and differ markedly from those obtained in Mediterranean countries and Iran, where geraniol frequently exceeds 20% and citronellol reaches up to 48% (Jaradat et al., 2022). Such geographic variations are mainly attributed to climatic and edaphic factors, confirming the existence of distinct chemotypes within the species.

The Algerian oil showed a lower geraniol content (5.35%) and relatively high levels of isomenthone and citronellyl esters, placing it within the so-called “African” chemotype (Fleisher and Fleisher, 1985; Verma et al., 2013). This profile is consistent with several recent studies reporting similar compositions dominated by oxygenated monoterpenes. For instance, Wei et al. (2022) reported citronellol (23.52%), geraniol (11.01%), geranyl tiglate (4.76%), and citronellyl formate (3.58%) as major compounds. Likewise, Dos Santos et al. (2024) reported citronellol (23.00%), cis-geraniol (21.33%), citronellyl formate (9.74%), and geranyl tiglate (3.46%). Comparable proportions were also observed by Atailia and Djahoudi (2015) — citronellol (19.22%), geraniol (14.03%), citronellyl formate (10.02%) and linalool (5.6%) — and by Lothe and Verma (2023) — citronellol (32.29%), geraniol (18.20%), and citronellyl formate (7.84%).

The high proportions of oxygenated monoterpenes (11.48%) and sesquiterpene hydrocarbons (11.41%) further support the strong antimicrobial, antioxidant, and enzyme-inhibitory potential previously documented for Maghreb oils. Evidence from GC-MS analyses consistently highlights monoterpenoid alcohols as the dominant fraction, with citronellol and geraniol as the most abundant constituents across regions, though proportions vary — higher geraniol in Mediterranean accessions versus higher citronellol in Maghreb oils — due to local climate and harvest stage (Al-Mijalli et al., 2022**;** Gusinac-Avdović et al., 2024). Inter-study discrepancies arise mainly for minor compounds, such as eudesmols, which are more prominent in North African samples (M'Hamdi et al., 2024).

### 4.2 Antifungal activity

PGEO demonstrated broad-spectrum antifungal activity against the six *Candida* species tested, with inhibition zone diameters (DZIs) ranging from 15.7 ± 0.8 mm (*C.krusei*) to 24.5 ± 1.2 mm (*C. albicans* ATCC 10231). The MIC values obtained for PGEO (0.312–2.5 mg/mL) and the universally fungicidal MFC/MIC ratio of 2 represent the central pharmacological findings of this study. The fungicidal criterion (MFC/MIC ≤ 4, CLSI) was met uniformly across all six *Candida* species, indicating that PGEO exerts a direct lethal effect rather than merely inhibiting fungal growth — a feature that distinguishes PGEO from fluconazole, which is inherently fungistatic at standard clinical concentrations. From a mechanistic perspective, this fungicidal activity is likely mediated by disruption of fungal cell membranes, leakage of intracellular contents, and interference with ergosterol biosynthesis, as previously demonstrated for monoterpene alcohols such as citronellol and geraniol (Bakkali et al., 2008**;** Nazzaro et al., 2017).

The clinical relevance of fungicidal activity in invasive candidiasis — where mortality reaches 40–50% in intensive-care patients — has been underscored by Pappas et al. (2018) and the WHO Fungal Priority Pathogens List (World Health Organization (WHO), 2022), which identify *C. albicans*, *C.tropicalis*, and *C.krusei* as priority targets requiring novel therapeutic strategies.

The susceptibility order established herein — *C.albicans* ATCC 10231 > *C.albicans* (clinical) ≈ *C. tropicalis* ≈ *C.lusitaniae* > *C.kefyr* > *C.krusei* is largely consistent with previously reported sensitivity patterns for geranium-derived oils. Boukhatem et al. (2013) demonstrated that Algerian PGEO exerted greater antifungal activity against yeasts than bacteria, with *C.albicans* being the most susceptible species tested. The MIC of 0.312 mg/mL for *C.albicans* ATCC 10231 is particularly competitive: Mahboubi et al. (2018) reported MICs of 1.06–1.48 µL/mL (≈ 0.93–1.30 mg/mL) for Iranian PGEO against clinical *C. albicans* isolates, while Hsouna and Hamdi (2012) measured MFCs of 0.039–0.625 mg/mL for Tunisian PGEO against *Candida* spp. Van Zyl et al. (2006) demonstrated that citronellol is the most fungicidally active constituent of geranium EO against *C. albicans* and *C.neoformans*, followed by geraniol — a hierarchy perfectly reflected by the major-compound composition of our PGEO (citronellol 28.68% > geraniol 5.35%).

The species-specific differences in PGEO sensitivity can be rationalized in terms of cell-wall and membrane composition, enzyme expression profiles, and drug-resistance mechanisms. *C. albicans* ATCC 10231 exhibited the highest susceptibility (MIC = 0.312 mg/mL; DZI = 24.5 mm), consistent with its ergosterol-rich membrane, which is highly accessible to monoterpene alcohols such as citronellol and geraniol (Sharma et al., 2016; Bakkali et al., 2008). The clinical *C. albicans* isolate showed slightly reduced susceptibility (MIC = 0.625 mg/mL), likely reflecting efflux-pump overexpression (CDR1/CDR2) or ERG11 mutations commonly observed in clinical settings (Whaley et al., 2017). *C. tropicalis* and *C. lusitaniae* showed intermediate MICs (0.625 mg/mL), consistent with sensitivity patterns previously reported for oxygenated monoterpene-rich essential oils (Hsouna and Hamdi, 2012). *C. kefyr* was more resistant (MIC = 1.25 mg/mL), which may reflect differences in membrane lipid composition or reduced expression of specific ergosterol-biosynthesis enzymes targeted by geraniol (Mahboubi et al., 2018). *C. krusei* (syn. *Pichia kudriavzevii*) was the least susceptible species (MIC = 2.5 mg/mL), consistent with its constitutive overexpression of Abc1p and Abc11p efflux pumps and reduced azole-class compound accumulation (Lamping et al., 2009; Pfaller et al., 2010). Nonetheless, PGEO retained fungicidal activity (MFC/MIC = 2) against this WHO Priority Pathogen, underscoring the advantage of its multi-target mechanism over single-target antifungals such as fluconazole.

The retained fungicidal activity against intrinsically fluconazole-resistant *C.krusei* (WHO Priority Pathogen) represents a clinically critical finding. Fluconazole’s mechanism of action via Erg11p/CYP51 (lanosterol 14α-demethylase) inhibition is inefficient against this species, which overexpresses membrane efflux pumps (Abc1p, Abc11p) and exhibits reduced azole affinity (Pappas et al., 2018**;** Whaley et al., 2017). PGEO’s multi-target mechanism likely underlies this retained activity, consistent with data from Karpiński et al. (2023) showing *Lamiaceae* EO activity against azole-resistant non-albicans *Candida*, and with the principle established by Touati et al. (2025) that the multi-target mechanism of essential oils intrinsically minimizes resistance selection pressure.

Comparing our findings with previously published antifungal studies on geranium essential oils and related monoterpene-rich preparations allows a clearer contextual positioning of PGEO activity. The MIC of 0.312 mg/mL for *C. albicans* ATCC 10231 obtained here is markedly lower than the 1.06–1.48 mg/mL (approximately 0.93–1.30 mg/mL) reported by Mahboubi et al. (2018) for Iranian PGEO against clinical *C. albicans* isolates, and within the range of 0.039–0.625 mg/mL MFCs documented by Hsouna and Hamdi (2012) for Tunisian PGEO against *Candida* spp. The lower MIC observed in our study likely reflects both the higher citronellol content (28.68% vs. 19–23% in previously cited Algerian oils) and the fungicidal nature of the activity confirmed by MFC/MIC = 2. Compared with studies on other essential oils active against non-albicans *Candida* species, Dzamic et al. (2014) reported MICs of 0.5–2.0 mg/mL for PGEO against dermatophytes and reference-strain *C. albicans*, results broadly consistent with our data. For *C. tropicalis* and *C. lusitaniae*, MIC comparisons with PGEO are scarcely available, reinforcing the novelty of our dataset for these clinically relevant non-albicans species. Regarding *C. krusei*, the MIC of 2.5 mg/mL obtained here is lower than the 4.0–8.0 mg/mL reported for thymol-rich or carvacrol-rich oils (Man et al., 2022), consistent with the superior tolerance of *C. krusei* to membrane-active agents but also with the distinct multi-compound composition of PGEO which may partially overcome single-mechanism resistance. Collectively, these comparisons position PGEO among the most active geranium-based essential oils reported for clinical-isolate *Candida* species, particularly for azole-resistant strains.

### 4.3 Antibiofilm activity

In the present study, PGEO exhibited potent antibiofilm activity, reducing biofilm biomass (crystal violet) by 48.67 ± 1.55% (*C.krusei*) to 97.80 ± 1.47% (*C.albicans* ATCC 10231) at MIC. The highest inhibition was observed against *C.albicans* ATCC 10231 (97.80 ± 1.47%) and *C.tropicalis* (82.10 ± 1.40%), markedly outperforming fluconazole, which achieved only partial inhibition against susceptible strains and 0% against *C.krusei* at all tested concentrations (**Figure 3**). Notably, PGEO retained 48.67 ± 1.55% inhibition against *C.krusei*, while fluconazole was completely inactive (0% at all concentrations), highlighting its therapeutic advantage in azole-resistant scenarios (Pappas et al., 2018**;** World Health Organization (WHO), 2022). These results surpass several *Lamiaceae* essential oils [60–85% inhibition at MIC; Karpiński et al. (2023)], and are superior to those previously reported for Algerian PGEO [72–89%; Boukhatem et al. (2013)].

The complementary XTT assay confirmed these findings, showing metabolic-viability inhibition ranging from 43.13 ± 1.22% to 82.10 ± 1.40% at MIC. The systematically lower XTT values relative to CV at equivalent concentrations (e.g., 82.10 ± 1.40% vs. 97.80 ± 1.47% for *C.albicans* ATCC 10231 at MIC) reflect the inherent methodological difference between the two assays: crystal violet quantifies total adherent biomass — including extracellular polymeric matrix (β-1,3-glucans, mannoproteins, eDNA) and dead cells — whereas XTT specifically measures mitochondrial dehydrogenase activity of viable sessile cells (Kuhn et al., 2003**;** Pierce et al., 2008). The persistence of stained biomass despite reduced metabolic activity therefore suggests that PGEO efficiently kills sessile cells while only partially dispersing the biofilm matrix.

The evaluation of antibiofilm activity across four concentrations (MIC to MIC/8) was designed to model pharmacokinetic gradients encountered in clinical practice: in topical antifungal formulations (creams, gels, oral rinses), drug concentrations decrease exponentially from the application surface toward underlying tissue layers, frequently reaching sub-MIC levels at the site of biofilm attachment (Clatworthy et al., 2007). The CV inhibition maintained at MIC/8 (6.33–18.40%) across all strains demonstrates that PGEO retains measurable antibiofilm efficacy across the full clinically relevant concentration range, not solely at the pharmacological peak (Kačániová et al., 2023).

At the molecular level, Gupta et al. (2021) demonstrated that geraniol eradicates *C.glabrata* biofilm by simultaneously targeting *Ras1/cAMP/PKA* signaling (inhibiting yeast-to-hypha transition), ergosterol biosynthesis, the plasma-membrane H^+^-ATPase, and *CDR1/CDR2* efflux pumps at sub-MIC concentrations — providing a coherent mechanistic basis for the polypharmacological antibiofilm action of PGEO observed here.

### 4.4 Inhibition of virulence-associated enzymes

PGEO displayed broad-spectrum antivirulence activity by simultaneously inhibiting four classes of extracellular hydrolytic enzymes.

#### 4.4.1 Phospholipase inhibition

PGEO significantly reduced phospholipase-associated activity, with the strongest effect observed against *C.albicans* ATCC 10231 (58%). Lower inhibition was detected for *C.krusei* (27%), suggesting species-dependent susceptibility. These results are in agreement with Costa et al. (2024) and Shariati et al. (2022), who reported that monoterpene-rich essential oils (geraniol and linalool) reduce phospholipase B (Plb1) secretion possibly through membrane perturbation. In contrast, *C.krusei* showed only moderate inhibition (27.00%; Pz = 0.584), consistent with its intrinsically lower basal phospholipase activity and reduced membrane susceptibility linked to altered ergosterol composition (Deorukhkar and Saini, 2013). Other studies have shown that *C.albicans* exhibits a high prevalence of phospholipase activity (93.3% of strains; Kantarcioglu and Yücel (2002)), whereas *C.parapsilosis* shows significantly lower phospholipase activity (16.08% of isolates; Pakshir et al. (2013)), supporting the species-dependent enzyme-inhibition patterns observed here.

#### 4.4.2 Secreted aspartyl protease inhibition

SAP inhibition reached 62.00% and 59.07% for *C.albicans* ATCC 10231 and the clinical isolate, respectively (Pz = 0.198–0.221). These values are superior to those reported by Mertas et al. (2015) for *Melaleuca alternifolia* essential oil (45–52%). The strong SAP inhibition is likely attributable to geraniol (5.35%) and citronellol (28.68%), which may impair SAP secretion through membrane integrity disruption rather than direct active-site binding (Sharma et al., 2016). The lower response in *C.krusei* (23.30%) may reflect species-specific differences in SAP expression profiles and cell-envelope permeability (Naglik et al., 2003).

#### 4.4.3 Esterase inhibition

Esterase was the least affected enzymatic activity (21.30–55.00%), confirming the general observation that fungal esterases are less sensitive to essential oils than phospholipases or SAPs (Ahaik et al., 2026). The lower inhibition observed in the clinical *C.albicans* isolate (34.47%) compared with the reference strain may result from overexpression of efflux pumps (Cdr1/Cdr2) in clinical isolates, as previously described by De Sousa et al. (2020).

#### 4.4.4 Hemolysin inhibition

Hemolysin inhibition was the most sensitive target, with rates ranging from 59.08% to 65.00% against *C.albicans* (both strains), *C.kefyr*, *C.lusitaniae*, and *C.tropicalis*. These values surpass those reported by Mogavero et al. (2022) and Hou and Huang (2024) for other terpene-rich oils, highlighting the particular efficacy of PGEO against hemolysin-mediated iron acquisition, a critical virulence factor in invasive candidiasis (Pina‐Vaz et al., 2004). Mechanistically, this strong inhibition may result from interference with the Ras1–Cyr1–PKA pathway that controls ECE1-encoded candidalysin expression and hyphal-associated hemolytic activity (Moyes et al., 2016).

#### 4.4.5 Integrated antivirulence perspective

PGEO displayed a broad-spectrum antivirulence action, simultaneously targeting four distinct classes of extracellular hydrolytic enzymes — a feature that distinguishes it from the majority of plant-derived essential oils reported in the literature, which are typically evaluated against only one or two virulence determinants (El-Baz et al., 2021**;** Kaskatepe et al., 2022). The differential enzyme-inhibition profiles observed across the six tested strains with *C.albicans* ATCC 10231 showing 58–65% inhibition across the four enzyme classes versus 21–27% for *C.krusei* — are consistent with recognized interspecific differences in hydrolytic enzyme expression: *C.albicans* is the predominant producer of extracellular phospholipases and SAPs among clinical *Candida* species, while *C.krusei* exhibits reduced enzymatic virulence, relying instead on intrinsic drug resistance and biofilm formation as primary pathogenic strategies (Pappas et al., 2018**;** Taff et al., 2013). The consistently lower susceptibility of *C.krusei* across all virulence endpoints — enzyme inhibition, antibiofilm activity, and MIC reflects this species’ well-documented broad tolerance to membrane-active agents, attributable to constitutive overexpression of efflux pumps and alterations in membrane sterol composition that reduce compound accumulation irrespective of chemical class (Lamping et al., 2009**;** Pfaller et al., 2010). Although the absolute enzyme-inhibition values obtained for PGEO did not reach those of reference inhibitors at equivalent concentrations, its simultaneous multi-target action at sub-lethal concentrations represents a pharmacologically distinctive antivirulence strategy: attenuating *Candida* pathogenicity without exerting lethal selective pressure reduces the probability of resistance emergence, an advantage particularly relevant for recurrent or prophylactic use (Touati et al., 2025**;** World Health Organization (WHO), 2022).

### 4.5 Cytotoxicity and selectivity index: safety profile

The CC_50_ of PGEO on HEK-293 cells, determined by four-parameter nonlinear regression across three independent experiments (n = 18 wells), was 3.99 ± 0.12 mg/mL — a single intrinsic value independent of fungal-strain context, validated by doxorubicin (10 µM; viability 8.50 ± 1.47%, > 90% cell death) and DMSO 1% negative controls (viability 96.8–98.2%; p > 0.05 vs. untreated cells). Per ISO 10993-5, PGEO is classified as non-cytotoxic at concentrations ≤ 2 mg/mL (International Organization for Standardization (ISO), 2009).

Selectivity indices (SI = CC_50_/MIC) revealed a species-dependent therapeutic window: 12.79 (*C. albicans* ATCC 10231, excellent), 6.38 (*C.tropicalis*, *C.albicans* clinical, *C.lusitaniae*; favorable), 3.19 (*C.kefyr*, moderate), and 1.59 (*C.krusei*, narrow), following the criteria of Elizondo-Luévano et al. (2023). The narrow SI for *C.krusei* reflects its elevated MIC (2.5 mg/mL) due to intrinsic azole-class tolerance rather than any unusual mammalian toxicity of the oil. These values compare favorably to phenol-rich essential oils (thymol, carvacrol; SI = 2–4) associated with greater cytotoxicity and local-irritation risk (Bravo-Chaucanés et al., 2022**;** Man et al., 2022), and are consistent with values reported for Algerian PGEO against cancer cell lines (Boukhatem et al., 2022).

These findings suggest that PGEO showed limited cytotoxicity at concentrations ≤2 mg/mL encompasses the MICs of the four most susceptible species (*C.albicans* ATCC: 0.312 mg/mL; clinical isolate, *C.lusitaniae*, *C.tropicalis*: 0.625 mg/mL). This safety margin is supported by published data with *P.graveolens* EO showing no cytotoxic effect on normal cells at ≤ 2 mg/mL, with selective activity against A549, MCF-7, and PC3 cancer lines; Jaradat et al. (2022) similarly reported selective cytotoxicity against MCF-7, Hep3B, and HeLa (IC_50_ 32–315 µg/mL) with minimal toxicity toward normal cells — a > 100-fold differential consistent with the higher cholesterol content of cancer-cell membranes enhancing monoterpene permeation (Al-Saffar et al., 2017**;** El-Garawani et al., 2019).

The mechanistic basis for mammalian selectivity lies in the ergosterol–cholesterol paradigm: citronellol (28.68%) and geraniol (5.35%) preferentially bind ergosterol, inducing membrane destabilization, increased fluidity, and ergosterol depletion in fungal cells while sparing cholesterol-rich mammalian membranes (Bravo-Chaucanés et al., 2022). Geraniol further modulates CaCdr1p efflux-pump activity (competitive inhibition), is synergistic with fluconazole without additional mammalian toxicity, and retains fungicidal activity against.

multidrug-resistant *Candida auris* — depleting ergosterol, inhibiting biofilm formation, and demonstrating *in vivo* efficacy in *Caenorhabditis elegans* infection models and enhanced macrophage-mediated killing in THP-1 cells (Touati et al., 2025).

Collectively, the SI values, the non-cytotoxic safety margin, and the multi-target mechanism of action — limiting resistance-emergence probability — position PGEO as a stronger topical or adjunctive candidate than phenolic-rich oils. Translation into *in vivo* pharmacokinetic and tolerability models remains warranted to establish a definitive systemic-safety profile.

## 5 Conclusion

This study demonstrates that *Pelargonium graveolens* essential oil from northeastern Algeria possesses significant fungicidal, antibiofilm, and multi-target antivirulence activities against relevant *Candida* species, including fluconazole-resistant strains. The oil effectively inhibited key virulence enzymes and biofilm formation at sub-inhibitory concentrations while maintaining a favorable selectivity profile toward normal HEK-293 cells (SI up to 12.79). These findings highlight the therapeutic potential of this Algerian geranium oil as a natural alternative or adjuvant for the management of biofilm-associated candidiasis. Future *in vivo* investigations and pharmaceutical formulation studies are recommended to translate these promising *in vitro* results into clinical applications.

## Data Availability

The original contributions presented in the study are included in the article/supplementary material, further inquiries can be directed to the corresponding author/s.

## References

[B1] Adams R. P. (2007). Identification of essential oil components by gas chromatography/mass spectrometry (Carol Stream, IL: Allured Business Media).

[B2] Adams R. P. Thomas P. Rushforth K. (2007). The leaf essential oils of the new conifer genus, Xanthocyparis: Xanthocyparis Vietnamensis and X. nootkatensis. J. Essent. Oil Res. 19, 30–33. doi: 10.1080/10412905.2007.9699223 37339054

[B3] Ahaik I. Nunez-Rodriguez J. C. Abelló-Cros S. Yanes O. Bouhdid S. Gabaldón T. (2026). Antifungal and anti-virulence activities of cinnamon, thyme, and clove essential oils against Candida species. Front. Pharmacol. 17, 1756267. doi: 10.3389/fphar.2026.1756267 41847137 PMC12989488

[B4] Al-Mijalli S. H. Mrabti H. N. Assaggaf H. Attar A. A. Hamed M. Baaboua A. E. . (2022). Chemical profiling and biological activities of Pelargonium graveolens essential oils at three different phenological stages. Plants 11, 2226. doi: 10.3390/plants11172226 36079608 PMC9459842

[B5] Al-Saffar A. Al-Shanon A. Al-Brazanchi S. Sabry F. Hassan F. Hadi N. (2017). Phytochemical analysis, antioxidant and cytotoxic potentials of Pelargonium graveolens extract in human breast adenocarcinoma (MCF-7) cell line. Asian J. Biochem. 12, 16–26. doi: 10.3923/ajb.2017.16.26

[B6] Amann V. Kissmann A.-K. Firacative C. Rosenau F. (2025). Biofilm-associated candidiasis: pathogenesis, prevalence, challenges and therapeutic options. Pharmaceuticals 18, 460. doi: 10.3390/ph18040460 40283897 PMC12030374

[B7] Asker A. Y. Al Haidar A. H. (2024). Cytotoxic properties of Pelargonium graveolens leaf extract and its green-synthesized gold nanoparticles (*in vitro* study). J. Taibah. Univ. Med. Sci. 19, 901–909. doi: 10.1016/j.jtumed.2024.03.011 39280190 PMC11393577

[B8] Atailia I. Djahoudi A. (2015). Chemical composition and antibacterial activity of geranium essential oil (Pelargonium graveolens L'Hér.) cultivated in Algeria. Phytothérapie 13, 156–162. doi: 10.1007/s10298-015-0950-2

[B9] Bakkali F. Averbeck S. Averbeck D. Idaomar M. (2008). Biological effects of essential oils–a review. Food Chem. Toxicol. 46, 446–475. doi: 10.1016/j.fct.2007.09.106 17996351

[B10] Benzaid C. Belmadani A. Tichati L. Djeribi R. Rouabhia M. (2021). Effect of Citrus aurantium L. Essential oil on Streptococcus mutans growth, biofilm formation and virulent genes expression. Antibiotics 10, 54. doi: 10.3390/antibiotics10010054 33429924 PMC7827172

[B11] Benzaid C. Tichati L. Djeribi R. Rouabhia M. (2019). Evaluation of the chemical composition, the antioxidant and antimicrobial activities of Mentha× Piperita essential oil against microbial growth and biofilm formation. J. Essent. Oil Bear. Plants 22, 335–346. doi: 10.1080/0972060X.2019.1622456 37339054

[B12] Boukhatem M. Sudha T. Darwish N. Nada H. Mousa S. (2022). Essence aromatique du Géranium Odorant (Pelargonium graveolens L’Hérit.) d’Algérie: Exploration des propriétés antioxydante, anti-inflammatoire et anticancéreuse (anti-angiogénique et cytotoxique), *in vitro* et in ovo, vis-à-vis de différentes lignées cellulaires cancéreuses métastasiques. Annales Pharmaceutiques Françaises ( Elsevier: Paris, France), 383–396. doi: 10.1016/j.pharma.2021.07.002 34310905

[B13] Boukhatem M. N. Kameli A. Saidi F. (2013). Essential oil of Algerian rose-scented geranium (Pelargonium graveolens): Chemical composition and antimicrobial activity against food spoilage pathogens. Food Ctrl. 34, 208–213. doi: 10.1016/j.foodcont.2013.03.045 38826717

[B14] Bravo-Chaucanés C. P. Vargas-Casanova Y. Chitiva-Chitiva L. C. Ceballos-Garzon A. Modesti-Costa G. Parra-Giraldo C. M. (2022). Evaluation of anti-Candida potential of Piper nigrum extract in inhibiting growth, yeast-hyphal transition, virulent enzymes, and biofilm formation. J. Fungi. 8, 784. doi: 10.3390/jof8080784 36012773 PMC9409899

[B15] Buzzini P. Martini A. (2002). Extracellular enzymatic activity profiles in yeast and yeast‐like strains isolated from tropical environments. J. Appl. Microbiol. 93, 1020–1025. doi: 10.1046/j.1365-2672.2002.01783.x 12452958

[B16] Clatworthy A. E. Pierson E. Hung D. T. (2007). Targeting virulence: a new paradigm for antimicrobial therapy. Nat. Chem. Biol. 3, 541–548. doi: 10.1038/nchembio.2007.24 17710100

[B17] Clinical and Laboratory Standards Institute (CLSI) (2018). Method for antifungal disk diffusion susceptibility testing of yeasts (Wayne, PA, USA: Clinical and Laboratory Standards Institute).

[B18] Clinical and Laboratory Standards Institute (CLSI) (2023). Reference method for broth dilution antifungal susceptibility testing of yeasts (Wayne, PA, USA: Clinical and Laboratory Standards Institute).

[B19] Costa P. D. Nogueira P. Nascimento Y. D. Sobral M. Silvestre G. Castro R. D. (2024). Bioactive potential of Eugenia luschnathiana essential oil and extract: antifungal activity against Candida species isolated from oncological paients. Braz. J. Biol. 84, e286419. doi: 10.1590/1519-6984.286419 39292142

[B20] Council of Europe (2023). Essential oils in herbal drugs (Determination of essential oils in herbal drugs). 11th ed (Strasbourg, France: European Directorate for the Quality of Medicines & HealthCare (EDQM).

[B21] Deorukhkar S. Saini S. (2013). Virulence markers and antifungal susceptibility profile of Candida glabrata: an emerging pathogen. Br. Microbiol. Res. J. 4, 39–49. doi: 10.9734/BMRJ/2014/5806

[B22] De Sousa L. V. N. F. De Oliveira Maia C. D. Carvalho I. S. Prata J. M. Arcanjo L. C. R. De Figueiredo Vieira M. . (2020). Candida albicans isolated from denture-related stomatitis in elderly patients: Antifungal susceptibility and production of virulence attributes. Exp. Result. 1, e43. doi: 10.1017/exp.2020.49 41292463

[B23] Dos Santos F. N. Fonseca L. M. Jansen-Alves C. Crizel R. L. Pires J. B. Kroning I. S. . (2024). Antimicrobial activity of geranium (Pelargonium graveolens) essential oil and its encapsulation in carioca bean starch ultrafine fibers by electrospinning. Int. J. Biol. Macromol. 265, 130953. doi: 10.1016/j.ijbiomac.2024.130953 38499124

[B24] Duarte M. C. T. Figueira G. M. Sartoratto A. Rehder V. L. G. Delarmelina C. (2005). Anti-Candida activity of Brazilian medicinal plants. J. Ethnopharmacol. 97, 305–311. doi: 10.1016/j.jep.2004.11.016 15707770

[B25] Dzamic A. M. Sokovic M. D. Ristic M. S. Grujic S. M. Mileski K. S. Marin P. D. (2014). Chemical composition, antifungal and antioxidant activity of Pelargonium graveolens essential oil. J. Appl. Pharm. Sci. 4, 001–005. doi: 10.7324/JAPS.2014.40301

[B26] El-Baz A. M. Mosbah R. A. Goda R. M. Mansour B. Sultana T. Dahms T. E. . (2021). Back to nature: combating Candida albicans biofilm, phospholipase and hemolysin using plant essential oils. Antibiotics 10, 81. doi: 10.3390/antibiotics10010081 33467766 PMC7830859

[B27] El-Garawani I. El Nabi S. H. Nafie E. Almeldin S. (2019). Foeniculum Vulgare and Pelargonium Graveolens essential oil mixture triggers the cell cycle arrest and apoptosis in MCF-7 cells. Anti-Cancer. Agents Med. Chem. 19, 1103–1113. doi: 10.2174/1573399815666190326115116 30914034

[B28] Elizondo-Luévano J. H. Rodríguez-Garza N. E. Bazaldúa-Rodríguez A. F. Romo-Sáenz C. I. Tamez-Guerra P. Verde-Star M. J. . (2023). Cytotoxic, anti-hemolytic, and antioxidant activities of Ruta chalepensis L.(Rutaceae) extract, fractions, and isolated compounds. Plants 12, 2203. doi: 10.3390/plants12112203 37299182 PMC10255231

[B29] Elyemni M. Louaste B. NeChad I. Elkamli T. Bouia A. Taleb M. . (2019). Extraction of essential oils of Rosmarinus officinalis L. by two different methods: Hydrodistillation and microwave assisted hydrodistillation. Sci. World J. 2019, 3659432. doi: 10.1155/2019/3659432 31057339 PMC6463580

[B30] Fleisher A. Fleisher Z. (1985). Yield and quality of essential oil from Pelargonium graveolens cultivated in Israel. J. Sci. Food Agric. 36, 1047–1050. doi: 10.1002/jsfa.2740361104 41531421

[B31] Floyd H. E. Kavanagh A. M. Lowe G. J. Amado M. Fraser J. A. Blaskovich M. A. . (2024). Standardisation of high throughput microdilution antifungal susceptibility testing for Candida albicans and Cryptococcus neoformans. Sci. Rep. 14, 23407. doi: 10.1038/s41598-024-74068-2 39379501 PMC11461513

[B32] Gaur P. Hada V. Rath R. S. Mohanty A. Singh P. Rukadikar A. (2023). Interpretation of antimicrobial susceptibility testing using European Committee on Antimicrobial Susceptibility Testing (EUCAST) and Clinical and Laboratory Standards Institute (CLSI) breakpoints: analysis of agreement. Cureus 15(3):e36977. doi: 10.7759/cureus.36977 37139290 PMC10149341

[B33] Giongo J. L. De Almeida Vaucher R. Fausto V. P. Quatrin P. M. Lopes L. Q. S. Santos R. C. V. . (2016). Anti-Candida activity assessment of Pelargonium graveolens oil free and nanoemulsion in biofilm formation in hospital medical supplies. Microb. Pathog. 100, 170–178. doi: 10.1016/j.micpath.2016.08.013 27544324

[B34] Gupta P. Gupta H. Poluri K. M. (2021). Geraniol eradicates Candida glabrata biofilm by targeting multiple cellular pathways. Appl. Microbiol. Biotechnol. 105, 5589–5605. doi: 10.1007/s00253-021-11397-6 34196746

[B35] Gusinac-Avdović Š.F. Mladenović M. Z. Radulović N. S. (2024). Essential-oil composition of plant species of the genus Pelargonium. Facta. Univ. Ser. Phys. Chem. Technol. 22, 61–77. doi: 10.2298/FUPCT2401061G

[B36] Hou G.-W. Huang T. (2024). Essential oils as promising treatments for treating Candida albicans infections: research progress, mechanisms, and clinical applications. Front. Pharmacol. 15, 1400105. doi: 10.3389/fphar.2024.1400105 38831882 PMC11145275

[B37] Hsouna A. B. Hamdi N. (2012). Phytochemical composition and antimicrobial activities of the essential oils and organic extracts from Pelargonium graveolens growing in Tunisia. Lipids Health Dis. 11, 167. doi: 10.1186/1476-511X-11-167 23216669 PMC3539951

[B38] International Organization for Standardization (ISO) (2009). Biological evaluation of medical devices — Part 5: Tests for *in vitro* cytotoxicity (Geneva, Switzerland: International Organization for Standardization).

[B39] Jaradat N. Hawash M. Qadi M. Abualhasan M. Odetallah A. Qasim G. . (2022). Chemical markers and pharmacological characters of Pelargonium graveolens essential oil from Palestine. Molecules 27, 5721. doi: 10.3390/molecules27175721 36080486 PMC9457828

[B40] Kačániová M. Vukic M. Vukovic N. L. ČmikovÁ N. Verešová A. Schwarzová M. . (2023). An in-depth study on the chemical composition and biological effects of Pelargonium graveolens essential oil. Foods 13, 33. doi: 10.3390/foods13010033 38201061 PMC10778218

[B41] Kantarcioglu A. Yücel A. (2002). Phospholipase and protease activities in clinical. Mycoses 45, 160–165. doi: 10.1046/j.1439-0507.2002.00727.x 12100532

[B42] Karpiński T. M. Ożarowski M. Seremak-Mrozikiewicz (2023). Anti-Candida and antibiofilm activity of selected Lamiaceae essential oils. Front. Biosci. (Landm. Ed.) 28, 28. doi: 10.31083/j.fbl2802028 36866556

[B43] Kaskatepe B. Aslan Erdem S. Ozturk S. Safi Oz Z. Subasi E. Koyuncu M. . (2022). Antifungal and anti-virulent activity of Origanum majorana L. essential oil on Candida albicans and *in vivo* toxicity in the Galleria mellonella larval model. Molecules 27, 663. doi: 10.3390/molecules27030663 35163928 PMC8838586

[B44] Kuhn D. Balkis M. Chandra J. Mukherjee P. Ghannoum M. (2003). Uses and limitations of the XTT assay in studies of Candida growth and metabolism. J. Clin. Microbiol. 41, 506–508. doi: 10.1128/jcm.41.1.506-508.2003 12517908 PMC149594

[B45] Lamping E. Ranchod A. Nakamura K. Tyndall J. D. Niimi K. Holmes A. R. . (2009). Abc1p is a multidrug efflux transporter that tips the balance in favor of innate azole resistance in Candida krusei. Antimicrob. Agents Chemother. 53, 354–369. doi: 10.1128/aac.01095-08 19015352 PMC2630665

[B46] Li S. Shen Z. Wang S. Peng Y. Qi W. (2025). Study on the impact of biofilm formation by Candida albicans in recurrent vulvovaginal candidiasis on drug susceptibility. Front. Cell. Infect. Microbiol. 15, 1663099. doi: 10.3389/fcimb.2025.1663099 41078368 PMC12507753

[B47] Li Y. Zhu W. Xiang Q. Kim J. Dufresne C. Liu Y. . (2023). Creation of a plant metabolite spectral library for untargeted and targeted metabolomics. Int. J. Mol. Sci. 24, 2249. doi: 10.3390/ijms24032249 36768571 PMC9916794

[B48] Ling Q. Zhang B. Wang Y. Xiao Z. Hou J. Xiao C. . (2022). Chemical composition and antioxidant activity of the essential oils of citral-rich chemotype Cinnamomum camphora and Cinnamomum bodinieri. Molecules 27, 7356. doi: 10.3390/molecules27217356 36364183 PMC9656011

[B49] Lothe N. B. Verma R. K. (2023). A study on geranium (Pelargonium graveolens L′ Herit ex Aiton) cultivars’ productivity and economics as intervening by diverse climatic conditions of the western peninsular region of India. Ind. Crops Prod. 200, 116882. doi: 10.1016/j.indcrop.2023.116882 38826717

[B50] Luo G. Samaranayake L. P. Yau J. Y. (2001). Candida species exhibit differential *in vitro* hemolytic activities. J. Clin. Microbiol. 39, 2971–2974. doi: 10.1128/jcm.39.8.2971-2974.2001 11474025 PMC88272

[B51] M'Hamdi Z. Bouymajane A. Riffi O. Filali F. R. Ettarchouch M. Elhourri M. . (2024). Chemical composition and antibacterial activity of essential oil of Pelargonium graveolens and its fractions. Arab. J. Chem. 17, 105375. doi: 10.1080/14786419.2024.2373961 38954513

[B52] Mahboubi M. Mahdizadeh E. Heidarytabar R. (2018). The anti-candidal activity of Pelargonium graveolens essential oils against clinical isolates of Candida albicans. Infectio 22, 9–12. doi: 10.22354/in.v0i0.698

[B53] Mahboubi M. Valian M. (2019). Anti-dermatophyte activity of Pelargonium graveolens essential oils against dermatophytes. Clin. Phytosci. 5, 25. doi: 10.1186/s40816-019-0121-3 38164791

[B54] Man A. Mare A.-D. Mares M. Ruta F. Pribac M. Maier A.-C. . (2022). Antifungal and anti-virulence activity of six essential oils against important Candida species–a preliminary study. Future Microbiol. 17, 737–753. doi: 10.2217/fmb-2021-0296 35531749

[B55] Mertas A. Garbusińska A. Szliszka E. Jureczko A. Kowalska M. Król W. (2015). The influence of tea tree oil (Melaleuca alternifolia) on fluconazole activity against fluconazole‐resistant Candida albicans strains. BioMed. Res. Int. 2015, 590470. doi: 10.1155/2015/590470 25722982 PMC4334616

[B56] Mogavero S. Höfs S. Lauer A. N. Müller R. Brunke S. Allert S. . (2022). Candidalysin is the hemolytic factor of Candida albicans. Toxins 14, 874. doi: 10.3390/toxins14120874 36548771 PMC9785678

[B57] Moyes D. L. Wilson D. Richardson J. P. Mogavero S. Tang S. X. Wernecke J. . (2016). Candidalysin is a fungal peptide toxin critical for mucosal infection. Nature 532, 64–68. doi: 10.1038/nature17625 27027296 PMC4851236

[B58] Naglik J. R. Challacombe S. J. Hube B. (2003). Candida albicans secreted aspartyl proteinases in virulence and pathogenesis. Microbiol. Mol. Biol. Rev. 67, 400–428. doi: 10.1128/mmbr.67.3.400-428.2003 12966142 PMC193873

[B59] National Institute of Standards and Technology (NIST) (2017). NIST/EPA/NIH mass spectral library (NIST 17). Gaithersburg:

[B60] Nazzaro F. Fratianni F. Coppola R. De Feo V. (2017). Essential oils and antifungal activity. Pharmaceuticals 10, 86. doi: 10.3390/ph10040086 29099084 PMC5748643

[B61] Negreiros M. D. O. Pawlowski Â. Zini C. A. Soares G. L. G. Motta A. D. S. Frazzon A. P. G. (2016). Antimicrobial and antibiofilm activity of Baccharis psiadioides essential oil against antibiotic-resistant Enterococcus faecalis strains. Pharm. Biol. 54, 3272–3279. doi: 10.1080/13880209.2016.1223700 27590861

[B62] Olascuaga-Castillo K. Castillo-Medina O. Villacorta-Zavaleta M. Diaz-Ortega J. Blanco-Olano C. Altamirano-Sarmiento D. . (2024). Extraction of essential oils by hydrodistillation of four aromatic species: Conditioning, extraction conditions, yield and chemical composition. Sci. Agropecu. 15, 385–408. doi: 10.17268/sci.agropecu.2024.029

[B63] Pakshir K. Zomorodian K. Karamitalab M. Jafari M. Taraz H. Ebrahimi H. (2013). Phospholipase, esterase and hemolytic activities of Candida spp. isolated from onychomycosis and oral lichen planus lesions. J. Mycol. Med. 23, 113–118. doi: 10.1016/j.mycmed.2013.04.007 23706304

[B64] Pappas P. G. Lionakis M. S. Arendrup M. C. Ostrosky-Zeichner L. Kullberg B. J. (2018). Invasive candidiasis. Nat. Rev. Dis. Primers 4, 18026. doi: 10.1038/nrdp.2018.26 29749387

[B65] Pfaller M. A. Diekema D. Gibbs D. Newell V. Ellis D. Tullio V. . (2010). Results from the ARTEMIS DISK Global Antifungal Surveillance Study 1997 to 2007: a 10.5-year analysis of susceptibilities of Candida species to fluconazole and voriconazole as determined by CLSI standardized disk diffusion. J. Clin. Microbiol. 48, 1366–1377. doi: 10.1128/jcm.02117-09 20164282 PMC2849609

[B66] Pierce C. G. Uppuluri P. Tristan A. R. Wormley F. L. Mowat E. Ramage G. . (2008). A simple and reproducible 96-well plate-based method for the formation of fungal biofilms and its application to antifungal susceptibility testing. Nat. Protoc. 3, 1494–1500. doi: 10.1038/nprot.2008.141 18772877 PMC2741160

[B67] Pina‐Vaz C. Gonçalves Rodrigues A. Pinto E. Costa‐de‐Oliveira S. Tavares C. Salgueiro L. . (2004). Antifungal activity of Thymus oils and their major compounds. J. Eur. Acad. Dermatol. Venereol. 18, 73–78. doi: 10.1111/j.1468-3083.2004.00886.x 14678536

[B68] Price M. F. Wilkinson I. D. Gentry L. O. (1982). Plate method for detection of phospholipase activity in Candida albicans. Sabouraudia. J. Med. Vet. Mycol. 20, 7–14. doi: 10.1080/00362178285380031 7038928

[B69] Ramage G. Borghi E. Rodrigues C. F. Kean R. Williams C. Lopez‐Ribot J. (2023). Our current clinical understanding of Candida biofilms: where are we two decades on? Apmis 131, 636–653. doi: 10.1111/apm.13310 36932821

[B70] Rüchel R. (1981). Properties of a purified proteinase from the yeast Candida albicans. Biochim. Biophys. Acta. Enzymol. 659, 99–113. doi: 10.1016/0005-2744(81)90274-6 7018586

[B71] Sadeghi I. Yousefzadi M. Behmanesh M. Sharifi M. Moradi A. (2013). *In vitro* cytotoxic and antimicrobial activity of essential oil from Satureja intermedia. Iran. Red. Crescent. Med. J. 15, 70. doi: 10.5812/ircmj.4989 23487431 PMC3589783

[B72] Saleh A. M. Aljada A. Rizvi S. A. Nasr A. Alaskar A. S. Williams J. D. (2014). *In vitro* cytotoxicity of Artemisia vulgaris L. essential oil is mediated by a mitochondria-dependent apoptosis in HL-60 leukemic cell line. BMC Complement. Altern. Med. 14, 226. doi: 10.1186/1472-6882-14-226 25002129 PMC4227289

[B73] Samaranayake Y. Dassanayake R. Jayatilake J. A. Cheung B. P. Yau J. Y. Yeung K. W. . (2005). Phospholipase B enzyme expression is not associated with other virulence attributes in Candida albicans isolates from patients with human immunodeficiency virus infection. J. Med. Microbiol. 54, 583–593. doi: 10.1099/jmm.0.45762-0 15888468

[B74] Schaller M. Borelli C. Korting H. C. Hube B. (2005). Hydrolytic enzymes as virulence factors of Candida albicans. Mycoses 48, 365–377. doi: 10.1111/j.1439-0507.2005.01165.x 16262871

[B75] Seddiki S. Boucherit K. Boucherit Z. (2010). Contamination des cathéters vasculaires et des sondes urinaires par des levures de Candida albicans prélevées des patients: formation des biofilms et résistance à l’amphotéricine B. MHA 22, 40–45.

[B76] Shariati A. Didehdar M. Razavi S. Heidary M. Soroush F. Chegini Z. (2022). Natural compounds: a hopeful promise as an antibiofilm agent against Candida species. Front. Pharmacol. 13, 917787. doi: 10.3389/fphar.2022.917787 35899117 PMC9309813

[B77] Sharma Y. Khan L. Manzoor N. (2016). Anti-Candida activity of geraniol involves disruption of cell membrane integrity and function. J. Mycol. Med. 26, 244–254. doi: 10.1016/j.mycmed.2016.04.004 27554866

[B78] Sierra G. (1957). A simple method for the detection of lipolytic activity of micro-organisms and some observations on the influence of the contact between cells and fatty substrates. Antonie. van. Leeuwenhoek. 23, 15–22. doi: 10.1007/BF02545855 13425509

[B79] Taff H. T. Mitchell K. F. Edward J. A. Andes D. R. (2013). Mechanisms of Candida biofilm drug resistance. Future Microbiol. 8, 1325–1337. doi: 10.2217/fmb.13.101 24059922 PMC3859465

[B80] Talapko J. Juzbašić M. Matijević T. Pustijanac E. Bekić S. Kotris I. . (2021). Candida albicans—the virulence factors and clinical manifestations of infection. J. Fungi. 7, 79. doi: 10.3390/jof7020079 33499276 PMC7912069

[B81] Touati A. Mairi A. Ibrahim N. A. Idres T. (2025). Essential oils for biofilm control: mechanisms, synergies, and translational challenges in the era of antimicrobial resistance. Antibiotics 14, 503. doi: 10.3390/antibiotics14050503 40426569 PMC12108346

[B82] Van Den Dool H. Kratz P. D. (1963). A generalization of the retention index system including linear temperature programmed gas-liquid partition chromatography. J. Chromatogr. 11, 463–471. doi: 10.1016/s0021-9673(01)80947-x 14062605

[B83] Van Zyl R. L. Seatlholo S. T. Van Vuuren S. F. Viljoen A. M. (2006). The biological activities of 20 nature identical essential oil constituents. J. Essent. Oil Res. 18, 129–133. doi: 10.1080/10412905.2006.12067134 37339054

[B84] Verma R. S. Rahman L. U. Verma R. K. Chauhan A. Singh A. (2013). Essential oil composition of Pelargonium graveolens L’Her ex Ait. cultivars harvested in different seasons. J. Essent. Oil Res. 25, 372–379. doi: 10.1080/10412905.2013.782476 37339054

[B85] Wei L. Yang H. Li H. Zhu M. Mi S. Lu Q. . (2022). Comparison of chemical composition and activities of essential oils from fresh leaves of Pelargonium graveolens L′ Herit. extracted by hydrodistillation and enzymatic pretreatment combined with a solvent-free microwave extraction method. Ind. Crops Prod. 186, 115204. doi: 10.1016/j.indcrop.2022.115204 38826717

[B86] Whaley S. G. Berkow E. L. Rybak J. M. Nishimoto A. T. Barker K. S. Rogers P. D. (2017). Azole antifungal resistance in Candida albicans and emerging non-albicans Candida species. Front. Microbiol. 7, 2173. doi: 10.3389/fmicb.2016.02173 28127295 PMC5226953

[B87] World Health Organization (WHO) (2022). WHO Fungal priority pathogens list to guide research, development and public health action.

